# Augmenting the Immune Response against a Stabilized HIV-1 Clade C Envelope Trimer by Silica Nanoparticle Delivery

**DOI:** 10.3390/vaccines9060642

**Published:** 2021-06-11

**Authors:** David Peterhoff, Stefanie Thalhauser, Jan M. Sobczak, Mona O. Mohsen, Christoph Voigt, Nicole Seifert, Patrick Neckermann, Alexandra Hauser, Song Ding, Quentin Sattentau, Martin F. Bachmann, Miriam Breunig, Ralf Wagner

**Affiliations:** 1 Institute of Medical Microbiology and Hygiene, Molecular Microbiology (Virology), University of Regensburg, 93053 Regensburg, Germany; christoph.voigt@stud.uni-regensburg.de (C.V.); nicole.seifert@uni-hohenheim.de (N.S.); patrick.neckermann@klinik.uni-regensburg.de (P.N.); alexandra.hauser@klinik.uni-regensburg.de (A.H.); 2 Institute of Clinical Microbiology and Hygiene, University Hospital Regensburg, 93053 Regensburg, Germany; 3 Institute of Pharmaceutical Technology, University of Regensburg, 93053 Regensburg, Germany; stefanie.thalhauser@gmail.com (S.T.); miriam.breunig@chemie.uni-regensburg.de (M.B.); 4 Department for BioMedical Research, University of Bern, 3010 Bern, Switzerland; jan.sobczak@dbmr.unibe.ch (J.M.S.); mona.mohsen@dbmr.unibe.ch (M.O.M.); martin.bachmann@dbmr.unibe.ch (M.F.B.); 5 Department of Immunology RI, University Hospital Bern, 3010 Bern, Switzerland; 6 EuroVacc Foundation, 1002 Lausanne, Switzerland; song.ding@eurovacc.org; 7 Sir William Dunn School of Pathology, University of Oxford, Oxford OX1 3RE, UK; quentin.sattentau@path.ox.ac.uk; 8 Jenner Institute, Nuffield Department of Medicine, University of Oxford, Oxford OX3 7DQ, UK

**Keywords:** HIV vaccine, silica nanoparticles, stabilized envelope trimer, Env

## Abstract

The delivery of HIV-1 envelope (Env) trimer-based immunogens on the surface of nanoparticles holds promise to promote immunogenicity with the aim of inducing a potent, durable and broad neutralizing antibody (bnAb) response. Towards that goal, we examined the covalent conjugation of Env to 100 nm and 200 nm silica nanoparticles (SiNPs) to optimize conjugation density and attachment stability. Env was redesigned to enable site-specific cysteine-mediated covalent conjugation while maintaining its structural integrity and antigenicity. Env was anchored to different sized SiNPs with a calculated spacing of 15 nm between adjacent trimers. Both particle sizes exhibited high in vitro stability over a seven-day period. After attachment, 100 nm particles showed better colloidal stability compared to 200 nm particles. Importantly, the antigenic profile of Env was not impaired by surface attachment, indicating that the quaternary structure was maintained. In vitro Env uptake by dendritic cells was significantly enhanced when Env was delivered on the surface of nanoparticles compared to soluble Env. Furthermore, multivalent Env displayed efficiently activated B cells even at Env concentrations in the low nanomolar range. In mice, antibody responses to nanoparticle-coupled Env were stronger compared to the free protein and had equivalent effects at lower doses and without adjuvant.

## 1. Introduction

The sequence diversity of HIV-1 poses a huge challenge to the development of a prophylactic vaccine [1,2]. Ideally, a vaccine should induce a long-lasting and potent neutralizing antibody response, that is able to neutralize the majority of circulating viral strains [3]. One possible route to this ultimate goal is the induction of broadly neutralizing antibodies (bnAbs). bnAbs naturally occur after a prolonged co-evolution in the infected host, and they recognize conserved epitopes of the HIV-1 envelope glycoprotein and interfere with its function as a viral membrane fusion machine [4,5]. Substantial progress has been made in the development of soluble and stabilized Env-based immunogens that provide an optimized antigenic profile and are recognized by several bnAbs, but not by non-neutralizing antibodies (nnAbs) [6]. However, immunization with these Env variants has so far failed to induce neutralization breadth, and moreover, the immunogenicity of the heavily glycosylated stabilized Env trimers is rather weak [7,8,9]. This begs the question, how can we boost immunogenicity and elicit the production of bnAbs.

A promising strategy is the presentation of Env antigens on the surface of nanoparticles in a multivalent array [10,11,12]. This is inspired by natural virus infection, as the immune system is geared to recognize microorganisms in the micro- to nanometer range exhibiting repetitive molecular patterns on their surface [13,14]. Nanoparticles mimic this phenomenon and support the shuttling of antigens to the lymph nodes, enhancing subsequent interactions with antigen presenting cells (APCs) and B cells. These interactions trigger the formation of germinal centers where somatic hypermutation of antibodies takes place [15,16,17]. It has been shown that the biophysical properties of the nanoparticles carrying Env immunogens are of particular importance and should be carefully considered to tailor the immunological processes towards the desired immune responses, including bnAb generation [18].

The size of the nanoparticles strongly influences trafficking from the periphery to the lymph nodes. It is widely accepted that nanoparticles between 20 and 200 nm are capable of trafficking within the lymphatics [19]. Within this range, smaller particles more efficiently accumulate within the lymph nodes [15]. In the context of HIV, it has recently been shown that a size of around 100 nm may be favorable for lymph node accumulation and delivery of the antigens to B cells and lymph node resident APCs [20]. Particulate antigens are efficiently recognized by APCs that in turn activate T helper cells, which provide essential costimulatory signals to B cells [21,22].

The multivalent display of Env trimers allows for high avidity interactions and crosslinking of several B cell receptors (BCRs), resulting in enhanced B cell activation [23,24]. Therefore, most approaches aim to maximize Env density on the particles; however, literature suggests an optimal center-to-center distance between two adjacent trimers to be in the range of 10–15 nm for enhanced B cell activation [25]. The interaction of Env and BCRs is only productive when the epitopes are intact and available, which makes the mode of Env attachment to the surface of nanoparticles a critical design issue. Only a site-selective attachment can control the orientation of Env trimers. Additionally, it is critical that the linkage between the Env-trimers and the nanoparticles is stable [18]. For example, linkages formed by non-covalent conjugation to liposomes via Ni-NTA suffered from rapid instability [26]. To enhance stability, covalent conjugation, via Michael addition for example, has been pursued and demonstrated clear superiority over non-covalent approaches. Furthermore, the stability of the particle platform itself should ensure that the particles reach the lymph nodes [23,25]. Finally, the particle chassis itself should be antigenically inert, and should at the same time exclusively support the antigenicity of the delivered antigen.

We have recently demonstrated that HIV Env trimers can be covalently conjugated to nonporous silica nanoparticles (SiNPs) in a site-specific manner that preserves their structural integrity and enhances the uptake of the delivered protein by antigen-presenting dendritic cells [27]. These Env trimers were based on the well-described BG505 SOSIP.664 sequence, which contained a C-terminal tag that provided a single cysteine for Michael addition-mediated coupling to SiNPs via a heterobifunctional crosslinker. The relatively low coupling yield of 90 trimers per particle, corresponding to 10% of the maximal possible trimer load, suggested a partial structural occlusion of the tag for efficient coupling. This raised concerns about the potential loss of the beneficial multivalent interactions in vivo.

Here, we re-designed Env to optimize the conjugation strategy. We used the aforementioned BG505 sequence and additionally adapted the modifications to a stabilized HIV-1 Clade C consensus variant (ConC) to show general applicability. We separated the coupling and purification tags, moving the coupling tag to the gp120 N-terminus and the purification tag to the gp41-ectodomain C-terminus. By introducing a pair of cysteines into the coupling tag, we supported preservation of the native disulfide topology of the protein. To improve spatial availability of the coupling tag during the conjugation process, we introduced a linker between the coupling tag and the Env sequence. Using the ConC variant, we optimized the conditions for efficient covalent conjugation of Env in a dense array to the surface of 200 nm SiNPs (SiNP_200_) as well as to smaller 100 nm SiNPs (SiNP_100_) to move closer to the optimal size for enhanced lymph node accumulation. We show efficient attachment of the optimized Env to differently sized SiNPs and characterize the particles regarding Env density, in vitro stability, and antigenicity. Furthermore, we demonstrate that the particles have enhanced interactions with dendritic cells and B cells compared to Env in its soluble form. Finally, we show that SiNPs enhance immunogenicity of Env in a mouse immunization study.

## 2. Materials and Methods

### 2.1. General Materials

All chemicals were purchased from Sigma Aldrich (Taufkirchen, Germany) unless otherwise stated. The 200 nm amine functionalized (4 µmol/g, 1.2 × 10^11^ particles/mg) silica nanoparticles and 100 nm particles functionalized with 1 µmol/g amino groups (9.5 × 10^11^ particles/mg, sicastar^®^ and sicastar^®^ greenF, 25 mg/mL in water) were supplied from micromod Partikeltechnologie (Rostock, Germany). FITC labeled anti-mouse monoclonal CD11c and APC-labeled anti-mouse monolonal CD11c, CD80, CD86, MHC-II, and the IgG isotypes were obtained from Miltenyi (Bergisch Gladbach, Germany). Env-specific monoclonal antibodies were purchased from Polymun Scientific (Klosterneuburg, Austria) or from the NIH HIV Reagent Program (Manassas, VA, USA).

### 2.2. Protein Expression Constructs

Expression constructs are based on the pcDNA™5/FRT/TO vector (Thermo Fisher, Waltham, MA, USA). Reference constructs encode a minimal version of the tissue plasminogen activator (mini-tPA) signal sequence (MDAMKRGLCCVLLLCGAVFVSPSAA) followed by the Env sequence and a C-terminal hexahistidine-tag (GS-H_6_). Variants optimized for cysteine-mediated coupling encode the mini-tPA signal sequence followed by the coupling tag (CAAC), a glycine-serine-linker ((G_4_S)_3_), the Env sequence and a C-terminal hexahistidine-tag (GS-H_6_). SOSIP-constructs encode the two cysteines for the formation of the SOS disulfide bridge (A501C, T605C) the IP exchange (I559P) and the optimized Furin protease cleavage site (R_6_) as described earlier [6]. The native flexible linker (NFL) constructs contain the same amino acid exchanges but the SOS disulfide bridge is omitted and the Furin protease cleavage site is substituted by a flexible linker ((G_4_S)_2_) as described before [28]. Env sequences were the stabilized BG505 sequence including the glycan-knock-in (T332N) to complete the 2G12-bnAb epitope [6] and a stabilized Clade C consensus sequence which bears the G473T exchange which eliminates CD4 binding [29]. This stabilized ConC trimer (originally referred to as ConCv5 KIKO) is the result of a stepwise stabilization process [30]. For simplicity, we refer to this Env trimer in this study just as ConC-SOSIP and ConC-NFL. As a control, a variant without an N-terminal tag was generated by deletion of the coupling tag and the N-terminal glycine-serine-linker. The plasmid encoding the *furin* gene was used as described earlier [27]. All constructs were optimized for human codon usage.

### 2.3. Antigen and Antibody Expression, Purification and Labelling

Env variants were produced and purified as described earlier [27]. Briefly, Expi293F cells were transfected using ExpiFectamine^TM^ according to the manufacturer’s recommendations. For SOSIP variants, co-expression of *furin* was conducted in a 1:3 ratio (*w*/*w*) of *furin* per *env*. Transfected cells were cultured for 5 days, and supernatants were harvested subsequently by centrifugation. Supernatants were sterile filtered (2 µm filter) and loaded onto HisTrap Excel columns (Cytiva, Marlborough, MA, USA). The column was washed with 20 mM imidazole in PBS and elution was performed with a linear imidazole-gradient in PBS from 20 mM to 400 mM imidazole. Fractions containing protein were pooled, and buffer was exchanged to PBS and applied to a Superdex 200 Increase 10/300 GL size exclusion column operated in PBS at a flowrate of 0.5 mL/min. Fractions from size exclusion chromatography (SEC) were analyzed by blue native-polyacrylamide gel electrophoresis (native PAGE) using ServaGel™ 4–16% gels (SERVA Electrophoresis, Heidelberg, Germany). Fractions containing trimer were pooled and concentrated to approximately 1 mg/mL.

Mature and germline antibodies as well as CD4-IgG, to the extent they were not acquired commercially or obtained via the NIH HIV Reagent Program (Manassas, VA, USA), were produced in-house in Expi293F cells as described above. Plasmids encoding heavy chain and light chain genes were transfected in a 1:1 ratio. Sterile filtered supernatants were loaded onto HiTrap MabSelect SuRe or HiTrap rProtein A FF columns (Cytiva, Marlborough, MA, USA) and washed with PBS. Antibodies were eluted by step elution with 100 mM glycine pH 3.2 and immediately buffer exchanged to PBS.

Labelling of antibodies was performed using the Alexa Fluor™ 647 (AF647) Antibody Labeling Kit (Thermo Fisher, Waltham, MA, USA). Protein concentrations were measured by a NanoDrop 1000 UV/Vis Spectrophotometer (Thermo Fisher, Waltham, MA, USA). For calculation of the degree of labelling (DOL), the protein concentration was determined by measuring the extinction at 280 nm (E_280_), the extinction at 650 nm (E_AF647_) and using the AF647-specific correction factor for spectral overlap (σ_280_ = 0.03) and the protein’s extinction coefficient (ε_protein_) according to Equation (1).

c_protein_ = (E_280_ − (E_AF647_ × σ_280_))/ε_protein_ (1)

Calculation of the DOL, corresponding to the number of dye molecules per protein molecule, was calculated according to Equation (2), where ε_AF647_ is the extinction coefficient of AF647 (239,000 cm^−1^ M^−1^).

DOL = E_AF647_/ε_AF647_ × c_protein_ (2)

### 2.4. Biophysical Characterization of the Antigens

For NanoDSF measurements on a Prometheus NT.48 (NanoTemper, Munich, Germany), the protein was diluted to a concentration of 0.075 mg/mL in PBS. NT.48 Grade Standard capillaries (NanoTemper, Munich, Germany) were filled with protein dilutions and measurements were performed with Prometheus NT.48. For thermal unfolding experiments, samples were heated with a constant linear heating rate of 1 °C/min from 20 °C to 95 °C and changes in tryptophan fluorescence upon solvent exposure were monitored at 330 nm and 350 nm. Melting temperatures (T_M_) were calculated from the partial derivative of the fluorescence intensity ratio at 350 nm and 330 nm (FI_350_/FI_330_) with respect to the temperature by the Prometheus NT.48 software.

### 2.5. Ni-Capture Enzyme-Linked Immunosorbent Assay Using Monoclonal Antibodies

Ni-capture Enzyme-Linked Immunosorbent Assay (ELISA) was used to analyze the antigenic profile of Env proteins. We added 350 ng of Env in 100 μL PBS into the wells of a 96-well preblocked Ni-NTA ELISA plate (Qiagen). Wells with PBS only served as background controls. After binding of the protein overnight at 4 °C, the plates were washed three times with Tris-buffered saline (TBS) and antibody was added in fourfold serial dilutions in PBS containing 2% skim milk starting at 80 nM. After 2 h incubation at room temperature, plates were washed six times with TBS before adding 50 μL of horse raddish peroxidase-conjugated polyclonal rabbit anti-human IgG secondary antibody (Dako, P0214, 1:5000 dilution in PBS containing 1% (*w*/*v*) BSA). Plates were incubated for 1 h at room temperature and subsequently washed six times with TBS. We Added 50 μL of Tetramethylbenzidine (TMB) substrate solution to the wells for 2 min and the reaction was stopped by adding 30 μL of 1 M sulfuric acid. Absorption was measured immediately at a wavelength of 450 nm in an ELISA plate reader (Microplate Reader Model 680, Bio-Rad, Hercules, CA, USA) in three technical replicates. The values were corrected by subtraction of their respective background controls and plotted against antibody concentration. Curves were fitted by applying a non-linear regression model (hyperbola) using GraphPad Prism 9.1.0 (GraphPad Software, San Diego, CA, USA).

### 2.6. Attachment of Env Variants to Silica Nanoparticles

The covalent attachment of Env variants to silica nanoparticles was performed as previously described [27]. The general procedure was conducted as follows. In brief, an amine to sulfhydryl reaction was performed using heterobifunctional linkers for Env conjugation to SiNPs. SiNP concentrations were adjusted based on the concentrations of stock suspensions provided by the manufacturer. For example, 25 mg/mL stock suspension was diluted with phosphate buffered saline (PBS, 150 mM, pH 7.4) to obtain a concentration of 12.5 mg/mL in 0.1 × PBS (15 mM, pH 7.4). Then, the particles were incubated with a molar excess of the respective linker for 1 h at room temperature. After separating unreacted linker by centrifugation (3 × 13,000 *g*, at 4 °C for SiNP_200_ particles and 2 × 20,000 *g*, at 4 °C for SiNP_100_ particles) particles were re-dispersed and Env trimers previously treated with 0.5 mM tris(2-carboxyethyl)phosphine (TCEP) were added at a 1:1 volume. For example, 180 µL of 1 mg/mL Env stock solution were added to 180 µL of 12.5 mg/mL particles corresponding to an Env to NH_2_ molar ratio of 1:12 for SiNP_200_ particles and a 1:3 molar ratio for SiNP_100_ particles. The reaction was incubated overnight at 4 °C, before an excess of mercaptoethanol was added to quench the reaction. Finally, the particles were washed twice by centrifugation and resuspended in PBS.

### 2.7. Characterization of SiNP Formulations

The size, polydispersity index (PDI) and zeta potential of the particles were determined using a Zetasizer Nano ZEN 3600 (Malvern, Herrenberg, Germany). Before measurement, particles were diluted to 0.1–0.5 mg/mL. Size and PDI of 200 nm blank particles were measured in water. All other samples were redispersed in 0.1 × PBS. Zeta potential was measured in 0.1 × PBS. Measurements were performed at least in triplicate and samples were analysed by the Malvern Zetasizer Software version 7.11 (Malvern Instruments, Worcestershire, UK).

The amount of attached Env was determined by a QuantiProTM BCA Assay Kit according to the manufacturer’s instructions. A standard curve was obtained in a range of between 1 µg/mL and 70 µg/mL derived from known concentrations of the protein stocks. Standards (*n* = 3) and samples (*n* = 4) were mixed with the copper (II) sulfate-containing working reagent. After incubation for one hour at 60 °C absorbance at 562 nm was read out with a plate reader (FluostarOmega, BMG Labtech, Ortenberg, Germany). The obtained concentration was then converted to the number of trimers per particle as previously described based on the particle number per mg provided by the manufacturer. Conjugation efficiency was determined by Equation (3) were Env_input_ is the amount of Env used for coupling and Env_coupled_ is the actually coupled amount of Env.

Conjugation efficiency (%) = Env_coupled_ (µg/mL)/Env_input_ (µg/mL) × 100% (3)

To confirm that Env trimers were successfully attached via covalent conjugation, sodium dodecyl sulfate (SDS)-polyacrylamide gel electrophoresis (PAGE) was performed. As controls, empty SiNPs, soluble Env, and Env simply mixed with SiNPs were applied. The Env concentration of each sample was adjusted to 2 µg, mixed with 4 × Laemmli sample buffer (sample buffer was supplemented with mercaptoethanol) and loaded on a 4.5% stacking and 12% separating acrylamide gel. After electrophoresis for 20 min at 90 V and 100 min at 120 V with decreasing current starting at 68 mA, gels were stained by Coomassie and visualized by a with a ChemiDoc™ MP gel documentation system (Bio-Rad Laboratories GmbH, Munich, Germany).

To evaluate in vitro stability, particle suspensions in PBS at 6.25 mg/mL concentrations were transferred to low bind tubes and incubated at 37 °C in a water shaking bath. At predetermined time points, the tubes were removed and centrifuged at 13,000× *g* for 10 min and at 20,000× *g* for 15 min, respectively, at 4 °C. Supernatants were removed and the particles were resuspended in fresh PBS. The samples were stored at 4 °C. Env amount in the supernatants was determined using a micro BCA assay.

### 2.8. Coupled Immunogen Characterization by Microscale Thermophoresis

Microscale thermophoresis (MST) was performed to obtain binding affinities of selected antibodies to the Env trimers immobilized on the nanoparticle’s surface. Representatively, the bnAbs VRC01 and PGT145 and the nnAbs F105 and 17b were used. Fixed concentrations of the labelled antibodies (1 nM) were added to a 1:1 (*v*/*v*) serial dilution of soluble Env (final concentrations: 0.006–200 nM), Env attached to SiNP_100_ particles (final concentrations: 0.006–200 nM) and to SiNP_200_ particles (final concentrations: 0.003–100 nM) in binding buffer (PBS supplemented with 0.05% polysorbate 20). Measurements were performed at 80% MST power and 15–20% LED power in standard capillaries on a Monolith NT.115 pico instrument (Nanotemper Technologies, Munich, Germany) at 25 °C. MST traces were recorded and analysed 1.5 s after the temperature jump as defaulted by the device. The fluorescence signal was plotted against Env concentrations and analyzed using the MO. Affinity Analysis v2.3 software (Nanotemper).

### 2.9. In Vitro Interactions with Bone Marrow Derived Dendritic Cells (BMDCs)

BMDC isolation and culturing was performed adapting a protocol previously reported [31]. Briefly, tibias and femurs of 29 and 32 week old male C57BL/J6 mice were used for the extraction of bone marrow. The obtained cell suspensions were cultured using VLE-RPMI 1640 medium (Biochrom GmbH, Berlin, Germany) supplemented with 10% heat-inactivated LT FBS, 50 µM 2-mercaptoethanol, 1% penicillin streptomycin (PAN-Biotech, Aidenbach, Germany) and 5 ng/mL GM-CSF (PeproTech, London, UK) at 37 °C and 5% CO_2_. After three and six days in culture, 10 mL fresh RPMI medium containing 5 ng/mL GM-CSF were added. After seven days, BMDCs were collected, washed and prepared for in vitro uptake and stimulation experiments.

### 2.10. BMDC Uptake and Stimulation

For uptake experiments, fluorescently labelled particles and Env previously labelled with Alexa Fluor 647 were used. To evaluate cellular uptake by flow cytometry, BMDCs were plated on a 96-well plate at a density of 3 × 10^5^ cells per well. Cells were cultured for 5 h and Env-carrying particles and soluble Env were added at final Env concentrations of 5 µg/mL. After incubation for 16 h, non-adherent and semi adherent cells were washed twice with PBS containing 5% fetal bovine serum (FBS) and finally resuspended in 0.5 mL PBS for the analysis with a FACSCanto II flow cytometer (BD, Franklin Lakes, NJ, USA). SiNP fluorescence was exited at 488 nm and Env fluorescence was excited at 633 nm. We acquired 10,000 events, and live cells were gated by forward and side scatter. The obtained data were analyzed using the Flowing software v2.5 (Turku, Finland).

Uptake was further analyzed by confocal microscopy. Cells were plated in glass bottom 8 well µ-slides (Ibidi, Planegg, Germany) at a density of 3 × 10^5^ cells per well and cultured for 5 h. Cells were treated for 16 h with soluble Env, and corresponding concentrations of Env immobilized on the particles’ surface. Cells were washed and covered with Leibowitz for the analysis with a Zeiss Axiovert 200 microscope combined with an LSM 510 laser-scanning device. Particles were excited with a 488 nm argon laser and detected with a 530/30 band pass filter. Env was excited simultaneously with a HeNe laser at 633 nm and recorded with a 650 nm long pass filter.

For stimulation experiments nanoparticles were prepared endotoxin-free. BMDCs were cultured at a density of 3 × 10^5^ cells per well in a 96-well plate for 5 h. Then, 4 µg Env either in its soluble form or attached to particles and corresponding amounts of blank particles were added. PBS was used as negative control and 1 ng/mL Lipopolysaccharide (LPS) as positive control. After an incubation time of 16 h, BMDC were washed and cells were incubated for 30 min with a FITC-labelled anti-CD11c antibody and either with APC labelled anti-CD80, anti-CD86 and anti-MHCII peptide monoclonal antibodies. After another washing step cells were resuspended in 0.5 mL PBS containing 5% FBS and surface marker expression was evaluated by a FACS Canto II flow cytometer (BD Biosciences, San Jose, CA, USA). We acquired 10,000 events, and live cells were gated by forward and side scatter. Gating of positive cells was then performed on isotype controls. Samples were analyzed using Flowing software (Turku Bioscience, Turku, Finland).

### 2.11. In Vitro Interactions with B Cells

To analyze B cell activation in vitro, Ramos B cells expressing the mature VRC01 B cell receptor were cultured and prepared at 4 × 10^6^ cells/mL. Cells were loaded with Fluo-4 Direct^TM^ calcium reagent (Thermo Fisher, Waltham, MA, USA) containing probenecid. After an incubation time of 45 min at 37 °C, cells were washed and resuspended in 2 mL RPMI-1640 for analysis with an Attune NxT Flow Cytometer (Thermo Fisher, Waltham, MA, USA). Blank SiNPs, soluble Env, and Env attached to SiNPs (Env concentration was adjusted to 25 nM, 12.5 nM, 6.25 nM and 1.25 nM) were each added to 200 µL cell suspension and Ca signals were measured by recording the Fluo-4 fluorescence for 5 min. To obtain a maximum calcium signal ionomycin was added at a final concentration of 1.3 µM to the cell suspension. Viable cells were gated via forward scatter and side scatter and kinetics were analyzed using FlowJo (Becton Dickinson, Franklin Lakes, NJ, USA).

For B cell receptor staining in confocal microscopy, cells were resuspended in PBS containing 1% BSA and loaded with FITC-labeled goat anti human IgM µ chain antibody at a final 1:10 dilution. After incubation for 30 min at 4 °C, cells were washed and stimulated with Env in its soluble form or covalently attached to SiNP_200_ particles and SiNP_100_ particles at final Env concentrations of 25 nM for 3 min. Cells were immediately washed with ice cold PBS, fixed with 4% paraformaldehyde and stained with DAPI. Confocal microscopy was conducted using a LEICA TCS SP5 confocal microscope. UV laser was used to detect cells and a 488 nm argon laser was used for excitation of FITC-labelled BCRs. Images were analyzed using Leica LAS X software. The experiment was performed once.

### 2.12. Mouse Immunization Experiments

The mouse experiments were conducted in strict compliance with the protocols approved by Swiss Federal Veterinary Office (license no. BE 70/18). At 8 weeks old C57BL/6J mice (Envigo RMS B.V., Venray, Netherlands) were immunized subcutaneously with 8 µg antigen either soluble, soluble mixed with 150 µg SiNPs or coupled to 150 µg SiNPs, in combination with 24 µg MPLA (Polymun Scientific, Klosterneuburg, Austria) in a total volume of 50 µL. For the low dose group, 2 µg Env coupled to 37.5 µg SiNPs were used for immunization and for the group without adjuvants, the volume of MPLA was substituted by PBS. Blood samples were collected from the tail veins of mice, directly into blood collection tubes (BD Microtainer, BD Life Sciences, NJ, USA). Tubes were subsequently centrifuged at 10,000× *g* for 90 s in order to obtain serum.

### 2.13. Analysis of Serum Reactivity and Avidity by ELISA

For analysis of the serum reactivity, Nunc MaxiSorp™ high protein-binding capacity 96-well ELISA plates (Thermo Fisher, Waltham, MA, USA) were coated with 50 µL PBS containing 3.5 µg/mL Env protein per well at 4 °C overnight. Plates were washed with Tris-buffered saline (TBS), and blocked with 200 µL buffer 1 (PBS, 1% I-Block (Thermo Fisher, Waltham, MA, USA) and 1x ROTI^®^Block (Carl Roth, Karlsruhe, Germany) for 30 min at ambient temperature. After washing with TBS, serial dilutions of mouse sera in 50 µL buffer 1 were applied for 1 h at ambient temperature. After washing with TBS, 50 µL peroxidase labelled anti-mouse IgG secondary antibody (Dako, P0260) at a 1:1000 dilution in TBS containing 1% BSA was added for 1 h at ambient temperature. After washing with TBS containing 0.05% Tween and an additional washing step with TBS, plates were developed with 50 µL in-house TMB substrate solution and the reaction was stopped with 25 µL 1 M H_2_SO_4_. Optical density at 450 nm (OD_450_) was measured immediately in an ELISA plate reader (Microplate Reader Model 680, Bio-Rad, Hercules, CA, USA) in three technical replicates.

Serum avidity was determined as previously described [32]. To this end, an additional step was implemented in the ELISA protocol. For this, after the serum binding step the plate was washed with TBS, and either 100 µL 1.5 M NaSCN or 100 µL BPS was added side by side for 15 min at ambient temperature. After washing with TBS, the protocol was continued as described above with the binding of the conjugate. The avidity index (AI) was calculated using the area under the curve (AUC) values after NaSCN-treatment (A_NaSCN_) and after PBS treatment (A_PBS_) using Equation (4).

AI = A_NaSCN_/A_PBS_ (4)

AUC values were calculated using GraphPad Prism 9.1.0 (GraphPad Software, San Diego, CA, USA).

### 2.14. Statistical Analysis

All data are indicated as means ± SD or medians. Statistical analysis was performed by using a one-way ANOVA combined with Šídák’s multiple comparisons test, or Welch’s ANOVA and the Dunnett T3 post-hoc test for pairwise comparisons using GraphPad Prism 9.1.0 (GraphPad Software, San Diego, CA, USA) in combination with IBM SPSS Statistics for Windows Version 26 (IBM Corp., Armonk, NY, USA). Significant differences were indicated as: * for *p* < 0.05, ** *p* < 0.01, *** *p* < 0.001 and **** *p* < 0.0001.

## 3. Results

### 3.1. Antigen Design and Biochemical and Biophysical Characterization

**Figure 1:**
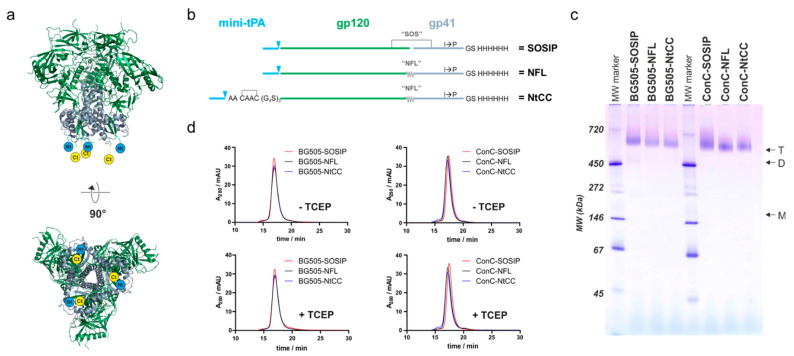
Design and characterization of Env variants used in this study. (**a**) Side view and base of the HIV envelope trimer (pdb code 4zmj). Gp120 colored in green, gp41 colored in grey, the N-terminus (Nt) and C-terminus (Ct) is symbolized in blue and yellow. (**b**) Topological representation of the variants of different sequences used in this study. Mini-tPA, minimized version of the tissue plasminogen activator signal peptide; triangles represent the cleavage site of the signal peptidase; SOS, gp120-gp41 disulfide bridge; NFL, native flexible linker; NtCC, N-terminal tag containing 2 cysteines; His_6_, hexahistidine tag. (**c**) Native PAGE of all variants used in this study with 2.5 μg of protein loaded on each lane of a 4–16% acrylamide native PAGE. Arrows indicate trimeric (T), dimeric (D) and monomeric (M) bands. MW marker indicates sizes of protein bands. (**d**) Analytic SEC profiles of all used Env variants with and without mildly reducing TCEP treatment. Env was incubated in absence (-TCEP) and presence (+TCEP) of 1 mM TCEP for 1 h at ambient temperature and incubated overnight at 4 °C. A total volume of 60 µL of a 0.4 mg/mL protein solution was loaded on a Superdex 200 Increase size exclusion column operated at a flow rate of 0.6 mL/min.

The N-terminus of HIV Env gp120 and the membrane proximal region of gp41 are both located at the membrane-directed base of the trimer’s ectodomain (Figure 1a). Thus, attachment via a reactive coupling tag located at either the N- or at the C-terminus of the truncated soluble gp41 ectodomain results in virus-like presentation of the trimer on the surface of a nanoparticle. To allow for independent modification, we decided to split the coupling and purification tag, and separately introduce them at the N- and C-termini of gp120 and gp41 ectodomains, respectively (topological representation in Figure 1b).

We speculated that a pair of cysteines separated by two alanines (CAAC) instead of a single cysteine per protomer, would better support native folding of the protein due to intra-tag disulfide bridge formation (Figure 1b). This would maintain the native disulfide-bridge topology of the rest of the protein and therefore support higher expression levels of well-folded protein. Hence, we introduced the CAAC sequence between a minimal version of the tissue plasminogen activator (mini-tPA) signal peptide and a (G_4_S)_3_-flexible-glycine-serine-linker at the N-terminus of the Env-coding sequence. As the mini-tPA signal will be removed after translocation into the endoplasmic reticulum, the N-terminus will consist of the CAAC sequence followed by a flexible linker for better accessibility during coupling to the nanoparticles.

For coupling via the thiols of the tag-encoded cysteines, the protein has to be treated with mild reducing reagents like TCEP. This poses the risk of reducing the SOS-disulfide-bridge which stabilizes the pre-fusion conformation of the soluble Env SOSIP constructs [6]. Therefore, we opted for a native flexible linker (NFL) between gp120 and gp41, which has been described as a versatile alternative to the reduction-sensitive SOS-disulfide-bridge [28]. To systematically evaluate our re-design, we generated three Env variants: (i) a SOSIP-version with a mini-tPA signal sequence at the N-terminus and a hexahistidine purification-tag at the C-terminus as a reference (SOSIP), (ii) the corresponding NFL-variant (NFL) and (iii) the NFL-variant with the N-terminal cysteine-containing coupling-linker (NtCC). To demonstrate general adaptability of these modifications, we generated the aforementioned three variants based on two sequences–the well-described BG505 SOSIP.664 sequence (BG505) as a reference and a stabilized Clade C consensus sequence (ConC, referred to as ConCv5 KIKO in [30]), which has further been engineered to avoid binding to CD4 (G473T, [29]).

The proteins were expressed in Expi293 cells (with co-expression of *furin* in the case of the SOSIPs) and purified from the supernatants via Ni-affinity purification. The yields of the corresponding variants were comparable and generally higher for the consensus variant (Table S1). After further purification via preparative size exclusion chromatography (SEC), the proteins were analyzed on a native PAGE, confirming a trimeric state of all variants with no apparent aggregation (Figure 1c). Cleavage status and correct formation of the SOS-disulfide bridge of the proteins was confirmed by applying the preparations to SDS-PAGE analysis under reducing and non-reducing conditions (Figure S1). To test the influence of mildly reducing TCEP-treatment on the trimeric structure, we performed analytical SEC runs and native PAGEs of TCEP-treated and untreated protein (Figure 1d and Figure S2). The trimeric structure was well preserved in all cases as apparent from the comparable elution profiles. Finally, the stability of the protein was assessed by nano-differential-scanning-fluorimetry measurements (Figure S3). BG505-NFL and BG505-NtCC show similar thermal unfolding transitions as compared to their SOSIP reference protein. ConC-SOSIP exhibits a 6 °C higher melting temperature (T_M_) as compared to the NFL-variants and a slightly less cooperative transition, whereas the coupling tag appears to have no influence on the thermostability as apparent from comparison of ConC-NFL and ConC-NtCC.

### 3.2. Antigenicity of the Soluble Env Trimers

**Figure 2:**
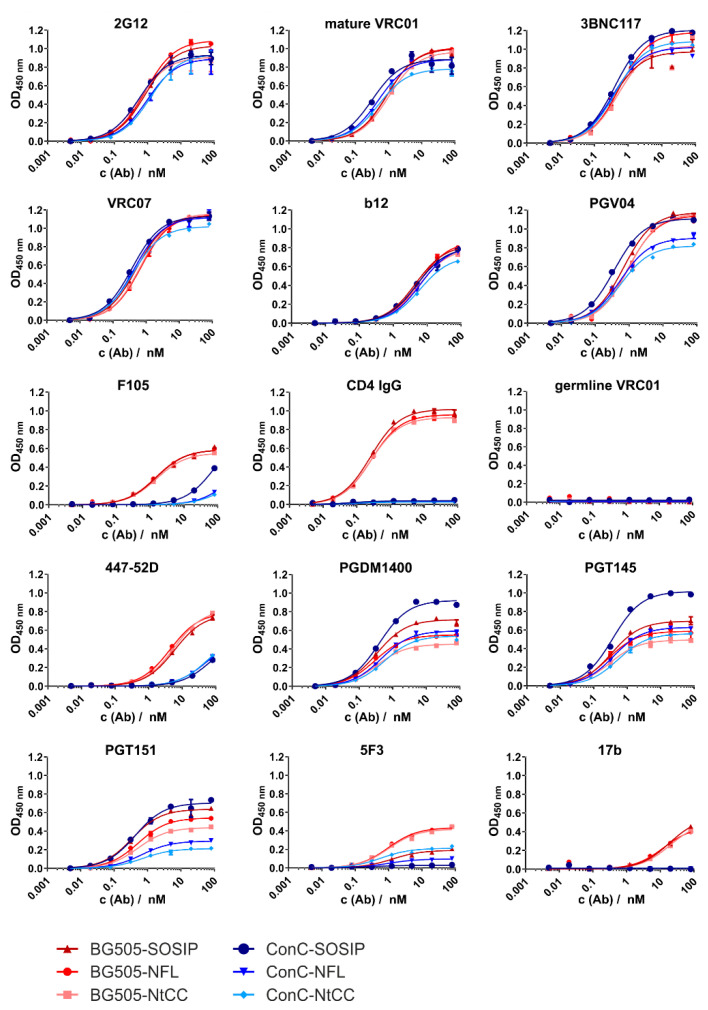
ELISA titration curves for all antibodies and variants. Absorption at 450 nm is plotted against the antibody concentration. We used 350 ng of Env per well for capturing of the trimer via its His_6_ tag onto the Ni^2+^-NTA surface. Measurements were conducted in duplicates of technical triplicates. Antibodies were titrated in fourfold dilutions starting at 80 nM.

We next analyzed the binding of a set of bnAbs and nnAbs directed to several prominent epitopes against all antigens to confirm structural integrity and optimal antigenicity (Figure 2). This was done in a Ni-capture ELISA format capturing the antigens via their His_6_-tags to preserve the trimeric structure and orient the antigen on the plate surface in a manner similar to how they will be displayed on the SiNP surface.

All variants showed comparable high signal intensities for the V3/N332 glycan supersite-directed antibody 2G12 which is minimally secondary structure-dependent. We tested several antibodies from different antibody classes directed to the CD4 binding site (CD4bs) supersite [33]. Comparable results were achieved for VRC01, 3BNC117, VRC07. Binding of CD4bs directed antibody b12 was comparable between all variants, but generally weaker than other CD4bs antibodies. CD4bs directed bnAb PGV04 showed strong binding with slight decreases in signal intensity for the ConC NFL variants. The signal intensities of the CD4bs nnAb F105 were very low for the ConC constructs, especially the NFL variants, and slightly higher for BG505 constructs. CD4-IgG showed strong binding to all BG505 variants and no binding to the ConC variants, as intended when introducing the G473T mutation. As expected, germline reverted VRC01 did not bind to any of the constructs, as our trimers do not feature the necessary adaptions of the CD4bs. Signal intensities for the hypervariable gp120 V3 loop directed neutralizing antibody 447–52D were intermediate for BG505 antigens, and low for ConC, suggesting pre-fusion state conformation of the trimer. Signal intensities of V2 apex directed bnAb PGDM1400 varied, but in general, the SOSIP references showed higher signals than the corresponding NFL variants. Similar results were determined for binding of the trimer dependent bnAb PGT145, but here the ConC-SOSIP-variant had a considerably higher signal intensity than all other variants. The Gp120-gp41-interface specific antibody PGT151 displayed variations regarding signal intensities between the constructs (approximately 0.2–0.7 OD for ConC-NtCC and ConC-SOSIP, respectively). Signal intensities for gp41 directed non-neutralizing antibody 5F3 were generally low, with NFL variants showing slightly increased binding. Similarly, the nnAb 17b, which binds to a CD4-induced conformational state only showed low affinity towards BG505 constructs and did not bind at all to ConC variants.

### 3.3. Coupling of the Protein to SiNPs and Characterization of the Conjugates

Due to its favorable expression yields, good folding, high stability, and preferential antigenicity, we decided to use the ConC-NtCC variant (from now on referred to as Env) in the subsequent experiments. Covalent attachment to nanoparticles was performed using aminated SiNP_200_ particles and SiNP_100_ particles via a site-selective two step amine-to-thiol reaction. Reaction conditions including the linker, the amount of Env, and the pH during the reaction were optimized to obtain optimal and efficient Env loading (Figures S4 and S5).

**Figure 3:**
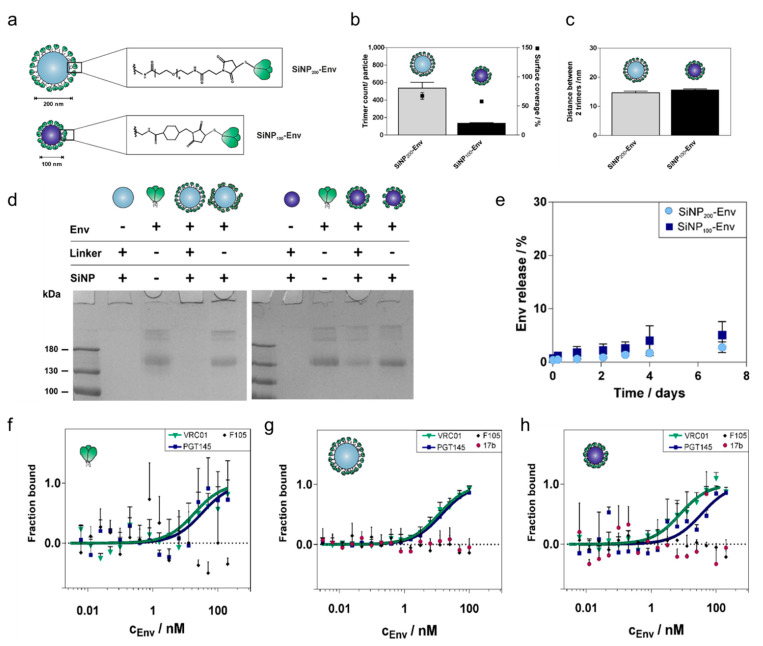
Coupling of Env to SiNP of different sizes and characterization of the conjugates. (**a**) The Env trimer (top) provides six cysteines in total and has an estimated square area of approximately 12 nm per side. Env was linked to SiNP_200_ particles via a SM(EG)_6_ linker (middle) and to SiNP_100_ particles via a sulfo-SMCC linker (bottom). (**b**) SiNP_200_ particles comprise around 580 trimers per particle after covalent conjugation corresponding to a surface coverage of about 79%. SiNP_100_ particles were loaded with about 130 trimers per particle corresponding to a surface coverage of about 60%. (**c**) Calculated center-to-center distances between two adjacent trimers were around 15 nm for both particle formulations. Results are presented as mean ± standard deviation of three independent experiments with quadruple measurements for each experiment (a total of twelve measurements for each sample). (**d**) Representative SDS PAGE of Env tethered to SiNP_1200_ particles (left) and to SiNP_100_ particles (right). Covalent conjugation to SiNP_200_ particles (left) and SiNP_100_ particles (right) was confirmed by SDS PAGE. The Env amount was adjusted to 2 µg for all samples. Soluble Env and Env adsorbed to plain SiNPs showed the characteristic gp140 band (lanes 3 and 5, left and right). Covalently conjugated Env stayed with the particles in the wells (lane 4, left and right). (**e**) Stability of Env attachment to the surface of nanoparticles. Release of Env trimers at 37 °C in PBS over seven days. Only a marginal amount of Env was released regardless of particle size. Results are presented as mean ± standard deviation (*n* = 3 samples). MST binding curves of the binding of VRC01, PGT145, F105, and 17b to (**f**) soluble Env, (**g**) SiNP_200_-Env and (**h**) SiNP_100_-Env. Results are presented as mean ± standard deviation (*n* = 6 measurements from two independent binding assays for soluble Env and SiNP_200_-Env; *n* = 3 measurements from one binding assay for SiNP_100_-Env).

Amine groups on the surface were activated with a heterobifunctional SM(EG)_6_ linker for the SiNP_200_ particles and a sulfo-SMCC linker for the SiNP_100_ particles. Then, Env was added at a 12:1 amine-to-Env molar ratio for SiNP_200_ particles and a 3:1 amine-to-Env molar ratio for SiNP_100_ particles, allowing for site-specific Michael addition between the malei-mide-carrying linkers and the thiols on the N-terminal cysteines of Env. In this way, two particle-Env conjugates with different sizes were generated (Figure 3b).

Different particle sizes lead to different maximum loading capacities. Based on the surface area of the respective particle and an estimated diameter of about 12 nm, one Env trimer would occupy a surface area of about 144 nm^2^. This results in a maximum loading of about 900 trimers per SiNP_200_ particle and a maximum loading of about 220 trimers per SiNP_100_ particle. Using optimized reaction conditions, about 580 Env trimers per particle were covalently conjugated to the surface of SiNP_200_ particles, which corresponds to 70% surface coverage (Figure 3b). Covalent conjugation of Env to SiNP_100_ particles resulted in about 130 trimers per particle and a surface coverage of about 60% (Figure 3c). As ligand density is a crucial parameter for interactions at the cellular level, Env spacing was calculated as well. Both formulations showed similar center-to-center distances of about 15 nm (Figure 3c).

Hydrodynamic diameters, polydispersity index (PDI), and zeta potential of the particles before and after trimer attachment are summarized in Table 1. After Env attachment, the particles increased in size. SiNP_200_ particles carrying Env showed sizes of about 390 nm and for SiNP_100_ particles about 130 nm. The large size and high PDI of SiNP_200_-Env suggest that the 200 nm SiNPs tend to aggregate after Env attachment. In contrast, SiNP_100_ particles were evenly distributed with a hydrodynamic diameter of about 130 nm and a PDI below 0.1. After Env conjugation, all samples carried a net negative surface charge.

To ensure that the trimers were indeed attached via covalent conjugation, denaturing reducing SDS PAGE was performed. As shown in Figure 3d, soluble Env shows a characteristic band at 140 kDa (both gels, lane 3). In contrast, SiNPs stayed in the gel loading wells, because their size restricts migration into the gel (both gels, lane 2). Thus, Env that is covalently conjugated via a stable thioether bond is expected to stay in the wells together with the particles. Covalent conjugation of Env to SiNP_200_ particles resulted in a stable linkage with no band visible in the gel (left gel, lane 4). For Env covalently attached to SiNP_100_ particles, a faint protein band was detected. Since equal amounts of soluble Env were used for conjugation, about 20% seem to be nonspecifically adsorbed to SiNP_100_ particles (right gel, lane 4). Env adsorbed to blank particles was included as a control. Env that was nonspecifically adsorbed to the particle surface migrated into the gel, indicated by a protein band at about 140 kDa (both gels, lane 5).

Stability of the particle conjugates in PBS at 37 °C was monitored over a seven-day period (Figure 3e). SiNP_200_-Env showed a low release of soluble Env of about 3%, while SiNP_100_-Env released about 5% of Env. In both cases, the majority of the trimers remained coupled to the surface of the nanoparticles.

Microscale thermophoresis (MST) measurements were performed to determine if the conjugation of Env to the nanoparticles affects the antigenic profile compared to soluble Env. Binding to selected bnAbs (VRC01 and PGT145) and nnAbs (F105 and 17b) was probed. VRC01 is directed against the CD4 binding site located laterally on the gp120 subunit, while the trimer-specific PGT145 binds to the trimer apex (V1V2 binding site). F105 binds to a conformational epitope that is dependent on CD4-induction, and 17b binds to the CCR5 or CXCR4 coreceptor binding site, which is likewise only exposed upon binding of CD4 to the trimer. The antigenic profile of soluble Env is shown in Figure 3e. Expectedly, both bnAbs efficiently recognized the antigen, while F105 did not show any binding. Comparable binding patterns were obtained when Env was attached to the surface of nanoparticles (Figure 3f,g). Particle size did not affect the binding efficiency of VRC01 to conjugated Env trimers. PGT145 showed a slight shift for Envs attached to SiNP_100_ particles. 17b and F105 were not able to bind the trimers indicating well-ordered trimers.

### 3.4. Uptake of Env-Decorated SiNPs by APCs

Antigen presenting cells (APCs) play a major role in the interplay of innate and adaptive immune responses. As dendritic cells are the most prominent APCs, we chose BMDCs as a relevant in vitro cell model to evaluate uptake of Env attached to SiNPs and subsequent stimulation. To investigate Env uptake, dendritic cells were incubated overnight with 5 µg of soluble Env or Env attached to SiNP_200_ (corresponding particle concentration: 0.2 mg/mL) and SiNP_100_ (corresponding particle concentration: 0.13 mg/mL). To track both Env and particle uptake, they were labeled with separate dyes. Uptake of Env was determined by flow cytometry. Env in its soluble form was taken up at a very low rate. Env attached to the nanoparticles was more efficiently taken up than soluble Env (Figure S6a,b). Comparing the two different nanoparticle sizes, Env delivered by SiNP_200_ particles displayed higher intracellular Env concentrations than Env delivered by the smaller SiNP_100_ particles. Cellular-associated fluorescence levels increased from soluble Env to SiNP_200_-Env. For example, mean fluorescence intensity (MFI) of SiNP_200_-Env (511 a.u.) was 2.4 and 13.1 times higher compared to SiNP_100_-Env (211 a.u.) and soluble Env (39 a.u.), respectively (Figure S6a). Similarly, the percentage of Env-positive cells increased from 30% for soluble Env to about 50% for SiNP_100_-Env up to about 70% for Env attached to SiNP_200_ particles. A comparable trend was observed for the percentage of SiNP-positive cells. SiNP_100_ particles were taken up by about 55% of the cells, while about 75% showed an uptake of SiNP_200_ particles (Figure S6c).

To confirm that Env and the particles were indeed taken up together, confocal microscopy experiments were performed. While soluble Env was not detectable within cells, Env trimers covalently attached to SiNPs of both sizes were localized within the cells. In addition, an overlay of the two channels revealed that Env was predominantly attached to the particles (Figure S6d).

To evaluate the influence of particulate Env delivery on the immune phenotype of BMDCs, immature BMDCs were stimulated with PBS, LPS, blank particles, soluble Env, SiNP_100_-Env, or SiNP_200_-Env. After 16 h, flow cytometry was used to evaluate the expression of CD11c, which represents the DC population, and of costimulatory molecules CD86, CD80, and MHC II peptide, which are involved in T helper cell activation. The percentage of the CD11c^+^ positive subset and the percentage of CD86^+^, CD80^+^ and MHC II peptide+ were analyzed (for representative flow cytometry plots refer to Figure S7). SiNP_100_-Env and SiNP_200_-Env significantly enhanced the amount of CD80-positive DCs compared to soluble Env. The amount of CD86- and MHC II peptide-positive cells slightly increased with increasing particle size, however differences were not significant (Figure S8). In addition, Env attached to particles did not show any upregulation of the selected surface markers per cell (data not shown).

### 3.5. In Vitro B Cell Activation by Env-SiNPs

**Figure 4:**
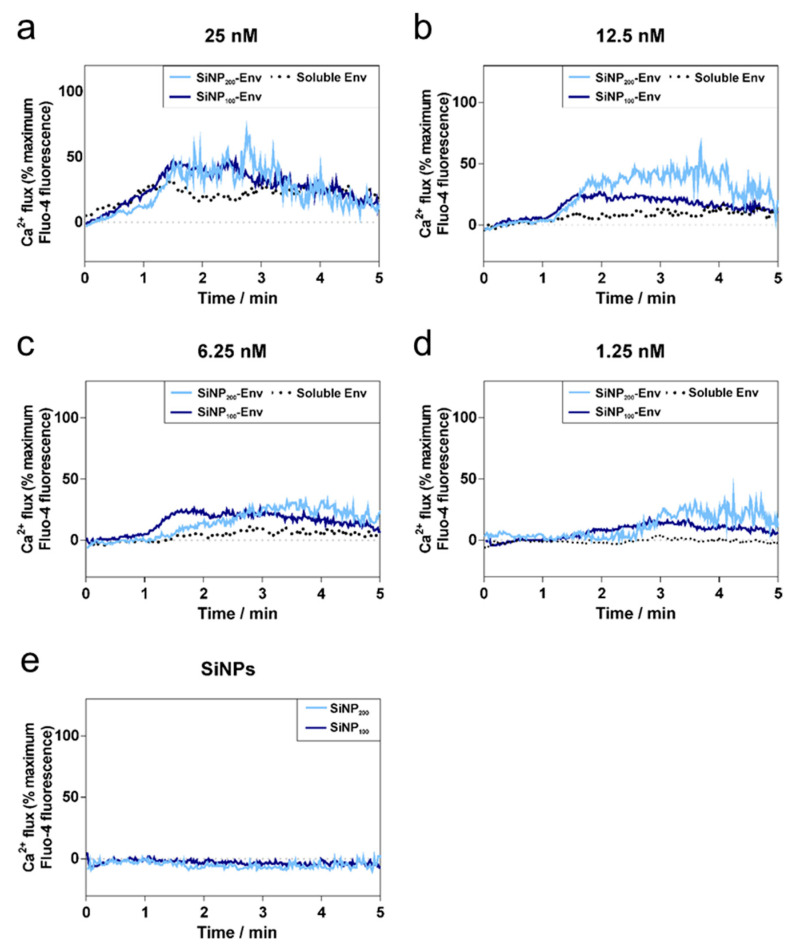
Calcium flux by VRC01-expressing B cells over time in response to decreasing concentrations of soluble Env, SiNP_100_-Env, and SiNP_200_-Env. (**a**–**d**) Activation of B cells at different protein concentrations of soluble or particle-bound Env. (**e**) SiNP_100_ particles and SiNP_200_ particles were used as controls and did not show any activation. Data shown is representative out of two separate assays.

Next, we evaluated whether Env attached to nanoparticles is able to interact with B cell receptors. Binding of antigens to the BCRs activates the B cells, resulting in a calcium flux into the cytosol. To this end, a B cell line expressing the mature VRC01 receptor on their surface were incubated with concentrations ranging from 25 nM to 1.25 nM of Env in its soluble form or with the same amount of Env attached to SiNPs. Intracellular calcium signals were monitored for 5 min by flow cytometry using a fluorescent calcium indicator. As shown in Figure 4a–d, Env immobilized on the surface of SiNPs via covalent conjugation more efficiently activated B cells, as indicated by a nearly doubled intracellular calcium signal compared to soluble Env. As expected, the maximum calcium signals decreased from about 50% to 25% with decreasing concentrations of Env for all formulations. While high concentrations of soluble Env showed activation as well (Figure 4a,b), at lower concentrations of Env, calcium signals were only detectable for Env attached to particles (Figure 4c,d). Comparing the nanoparticle sizes, SiNP_200_ particles showed higher peak values than SiNP_100_ particles. In addition, particularly at lower concentrations, SiNP_100_ particles showed a faster activation of B cells than SiNP_200_ particles. SiNPs were used as a negative control, and as shown in Figure 4e, particles alone did not activate B cells.

**Figure 5:**
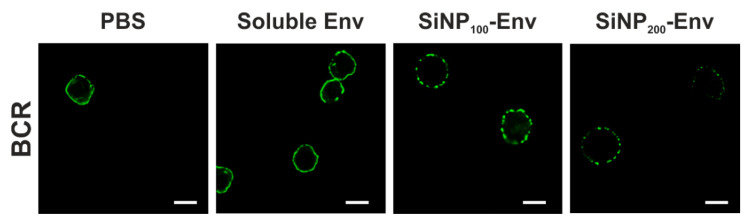
Representative confocal microscopy images of B cells incubated with PBS, soluble Env, Env covalently conjugated to SiNP_100_ particles and Env attached to SiNP_200_ particles. Green fluorescence indicates B cell receptors (scale bar = 10 µm).

For a better understanding of the underlying mechanisms of B cell activation, confocal microscopy studies were conducted. BCRs were stained with a FITC-labelled anti IgM antibody to visualize the VRC01 BCRs on the surface of the B cells. B cells were stimulated with PBS, soluble Env, or Env attached to SiNPs. As shown in Figure 5, stimulation with soluble Env or PBS resulted predominately in an even distribution of BCRs on the cell membrane. In contrast, applying Env attached to nanoparticles resulted in a clustered distribution of BCRs indicated by the dotted fluorescence.

### 3.6. In Vivo Qualification of SiNP_100_-Env-Conjugate Immunogenicity in Mice

**Figure 6:**
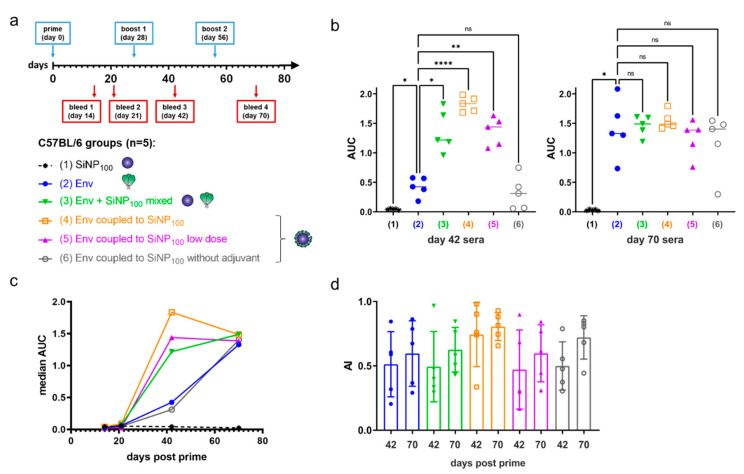
Immunization of C57BL/6J mice. (**a**) Immunization regimen and groups of the homologous prime–boost experiment. Normal dose groups received 8 µg protein, and low dose groups received 2 µg. Immunogens were adjuvanted at a 1:3 ratio of Env/MPLA except for the non-adjuvanted group; (**b**) Area under the curve (AUC) values of the ELISA serum titrations of each mouse, grouped per immunization group for bleed 3 (day 42) and bleed 4 (day 70). The median for each group is given and analyses were by one-way analysis of variance (levels of statistical significance are indicated as * *p* < 0.05, ** *p* < 0.01 and **** *p* < 0.0001); (**c**) The median AUC of each mouse group over the time span of the experiment; (**d**) Aligned dot plot showing mean and standard deviation of the avidity index of the later time points of mouse groups 2–6.

Next, we performed an immunization experiment in C57BL/6J mice. We used SiNP_100_ particles due to their favorable colloidal stability, their strong activation of VRC01-expressing B cells in vitro, and their potential of accumulating within B cell rich regions in the lymph node. Groups of five mice were immunized subcutaneously (s.c.) in a homologous prime-boost regimen with SiNP_100_, Env (soluble), a mixture of both (non-coupled), SiNP_100_-Env (coupled), and SiNP_100_-Env at a lower dose with and without monophosphoryl lipid A (MPLA) adjuvant, (Figure 6a). Immunization doses were 8 µg of protein mixed with 24 µg MPLA (1:3 ratio) per immunization for all groups except the low dose group, which was immunized with 2 µg, the non-adjuvanted group which received 8 µg protein without adjuvant, and the SiNP_100_ group which received 150 µg SiNP_100_ adjuvanted with 24 µg MPLA. Immunizations were performed at 4 week intervals and bleeding of the mice was scheduled 2 weeks after each immunization and at an additional 3 week time point after the prime as shown in Figure 6a. Autologous IgG responses against Env were measured in ELISA serum titrations and area under the curve (AUC) values were analyzed, since these values optimally reflect quality and quantity of the antibody response (for a comparison between EC50 and AUC values see Figure S9). Expectedly, the serum reactivity was low after the prime and increased after the first boost. Significant differences between the different groups after the first boost were partially compensated by the second boost (Figure 6b). The time course of the serum reactivity clearly demonstrates the superiority of the SiNP_100_-Env formulation (Figure 6c, median per group). As a measure for B cell maturation, we analyzed the avidity of the sera for the later time points. Differences were not significant within and between the groups, but a trend for a generally higher avidity after the second boost and for the SiNP_100_-formulation groups is observed (Figure 6d).

## 4. Discussion

Strategies to improve the immunogenicity of recombinant Env-based antigens by nanoparticulate delivery have received great attention in the last decade. By exploiting cues from nature, nanoparticles may improve trafficking to and within the lymph nodes while also presenting Env to APCs and B cells in a multivalent fashion. Several particle carrier types including virus-like particles (VLPs), liposomes, lipid nanocapsules, and iron oxide nanoparticles have been used to prove the superiority of surface-bound Env compared to Env in its soluble form in terms of B cell activation and antibody titers [12]. However, follow-up studies did not consistently corroborate these trends. One potential reason for this failure is the in vivo instability of liposomes [34]. Learning from these studies, the focus here was to improve critical design criteria including particle type and stability, Env attachment and colloidal stability, nanoparticle size, Env orientation, and Env density [18].

In this study, we developed nanoparticle-Env conjugates which fulfill these requirements. To this end, we started with optimizing Env for efficient coupling to the nanoparticles. This was addressed by introducing an N-terminal tag featuring two cysteines in a configuration that allows the formation of a disulfide bridge and a glycine-serine-linker that improves the availability of the tag. We decided to separate the purification tag and coupling tag to allow for the necessary removal of the purification tag at a later stage of vaccine development without manipulating the properties of the coupling tag. This approach combined with the replacement of Env’s Furin cleavage site with a native flexible linker greatly increased the yields of well-folded trimer. This was true for both the well-studied BG505 as well as a stabilized and further optimized Clade C consensus sequence ConC, as proven by biochemical and biophysical methods and by measuring the binding profiles of the tagged and nontagged variants against a panel of bn- and nnAbs.

In the ELISA titration experiments, the corresponding variants of each characterized sequence show only minor differences. Pronounced differences were only seen when comparing the isolates and assessing the binding of nnAbs and CD4-Ig. The latter was expected, as the ConC variant contains the recently published mutation (G473T) which abrogates binding to CD4 [29]. The differences in binding of cleavage-sensitive bnAb PGT151 may be caused by the NFL, which has been shown to hinder binding of gp120/gp41 interface-directed antibodies [28,35]. Impaired binding of 5F3 may also reflect steric hindrance by the NFL, and also isolate-specific sequence differences.

Next, we optimized the attachment of ConC-NtCC Env to the two different sizes of nanoparticles with the goal of increasing linker density. Our previous approach resulted in a rather low Env density with distances of about 40 nm between two adjacent trimers [27]. Although our data suggested the superiority of these particles compared to adsorbed Env regarding in vitro stability and uptake by APCs, the activation of B cells is ultimately caused by a dense array of Env allowing for multivalent interactions and crosslinking of BCRs. Thus, optimal ligand spacing is therefore of paramount importance, even though it is difficult to define. The common “more than necessary” principle may not be generalized across different applications [36,37]. Consequently, adjusting the ligand density has to be empirically determined for each vaccination strategy. In the context of particulate Env delivery, Env spacing of about 14 nm has been reported to enhance antibody titers compared to soluble Env [25]. To achieve such a dense Env spacing, we used a new Env variant equipped with two cysteines at the N terminus, for site-selective covalent conjugation to the particle surface via Michael addition. Applying Env without an N-terminal cysteine tag to activated SiNP_200_ particles showed only low levels of attached Env (70 trimers per particle, Figure S10), indicating that the linkage was indeed formed via the reaction between the maleimide and the thiols. Optimizing reaction conditions resulted in a spacing of about 15 nm between two adjacent Env trimers for both particle sizes, which is in the required range.

Colloidal stability strongly influences the fate of nanoparticles within the body [38]. Silica particles often struggle with aggregation in biological media [39]. However, large aggregates most likely persist in the periphery and never reach the lymph nodes. In addition, aggregation would occlude the surface-bound Env and prevent interactions with important receptors. Our results indicated that SiNP_200_ particles tend to aggregate after Env attachment, although interactions with cells have not shown any limitations, which suggests that protein in the cell media may stabilize the particles. In contrast, SiNP_100_ particles showed excellent colloidal stability with a low polydispersity. In addition, the SiNP_100_ particles were stable over a period of eight weeks when stored at 4 °C (Figure S11).

Besides colloidal stability, the stability of both the particle platform and the linkage between Env trimers and nanoparticles are equally important to ensure that Env reaches the lymph nodes together with the particles. Both particle sizes were stable in physiological conditions and showed only marginal release of Env after one week. Although SDS PAGE showed that a small proportion (20%) of Env was nonspecifically adsorbed to SiNP_100_ particles, only 5% of Env was released. This suggests that Env is adsorbed via relatively strong interactions. In contrast, when Env was simply mixed with SiNP_200_ particles, about 30% of the attached trimers were released in this time (Figure S12). This also substantiates the superiority of covalently-coupled trimers over non-covalently conjugated trimers.

To assess the antigenic profile of Env attached to the particles, a set of antibodies consisting of bnAbs (VRC01, PGT145) and nnAbs (F105, 17b) was selected. These antibodies bind to defined epitopes, so their binding profile allow us to estimate the structural integrity and accessibility and, indirectly, the orientation of Env after attachment to the particle surface. The high Env density on the surface most likely enables bivalent binding of both Fab fragments of the antibodies. This may preclude a straightforward K_D_ fit, which is based on the law of mass action of a 1:1 monovalent interaction. Hence, MST was used to determine the binding patterns of attached Env and qualitatively evaluate trimer integrity and epitope accessibility. Binding patterns of soluble Env and Env attached to SiNP_200_ particles were nearly identical. However, binding of PGT145 to SiNP_100_ particles showed a small shift. This may be accounted for by the proportion of nonspecifically adsorbed trimers that may be oriented in a way that marginally impedes binding of PGT145. Since F105 and 17b did not show any binding, we estimate that the trimers are indeed well-ordered after coupling to SiNP_100_ particles.

The generation of antibodies requires a fine-tuned interplay between T cells and B cells. The activation of B cells strongly relies on the help provided by activated T helper cells in the form of costimulatory signals (e.g., cytokines and CD80-/CD86-binding) [15]. Therefore, interactions with APCs (particularly with DCs, which are the key cellular player in T cell activation) and interactions with B cell receptors are critical steps in B cell activation.

In terms of interactions with dendritic cells, our data revealed that particulate delivery of Env was necessary for internalization of Env by BMDCs. It is widely accepted that particulate antigens are more efficiently recognized by immune cells than their soluble counterparts [16]. The size of the nanoparticles strongly influences the mechanism and extent of cellular uptake [40]. The particles used for Env delivery in this work also showed size-dependent uptake, and Env internalization was increased when delivered with the larger particles. This is consistent with literature that reported that particles larger than 200 nm are more efficiently taken up by APCs than smaller ones [19]. Following uptake, antigens are processed by the DCs for subsequent presentation to T cells. Confocal microscopy demonstrated that Env was still attached to the particles and most likely located in lysosomal compartments as indicated by the punctuated spots. It has been shown that particles may prolong the presence of antigens in the cells by interfering with lysosomal processing [35]. Upon lysosomal processing, antigens are most likely loaded onto MHC II peptides for future priming of CD4^+^ cells. While recognition and uptake of antigens by DCs is required for T helper cell activation, processing by DCs may alter the bnAb epitopes from their native form, which could prevent appropriate interactions with BCRs [14]. Hence, moderate uptake, as obtained for SiNP_100_ particles might be a good compromise that allows a T helper cell-dependent B cell response.

Although Env delivered by nanoparticles exhibited a prolonged presence of Env within distinct compartments of DCs, this was not reflected in an upregulation of surface markers. Only particulate Env caused an increase in the amount of activated DCs. This low activation level is in line with previous studies delivering ovalbumin on the surface of mesoporous nanoparticles to BMDCs [41]. Hence, the low stimulation was not unexpected, and the addition of certain adjuvants capable of tailoring and boosting immune responses may be helpful. Here, nanoparticles offer an additional advantage, because they can be equipped with certain adjuvants to simultaneously deliver antigen and adjuvant to the APCs [42]. Overall, the increased cellular uptake that has been demonstrated for particle-bound Env is an essential prerequisite to accumulate antigen and adjuvant within DCs. Besides cellular uptake the intracellular processing of antigens also plays an important role in DC stimulation, but both events are not distinguished by our readout.

In terms of B cell activation, it was demonstrated that the densely arrayed Env trimers enhanced the activation of B cells expressing the VRC01 receptor compared to soluble Env. The first event of B cell activation is the binding of Env to the BCRs. Upon binding, intracellular phosphorylation of tyrosine kinases activates several signaling pathways including calcium flux into the cytosol [43]. The extent of B cell activation and resultant intracellular calcium signals is determined by several factors including antigen concentration, affinity and valency [43]. Soluble Env in its trimeric form has three VRC01 binding sites. However, due to spatial restrictions, it is likely that only monovalent binding occurs [44]. Nevertheless, affinity was apparently sufficient to activate B cells via monovalent binding at concentrations between 25 nM and 6.25 nM. In contrast, the attachment of Env on the particle surface enables multivalent Env display and hence, an increased avidity for the BCRs. This in turn may result in BCR crosslinking, which further triggers activation, as indicated by increased intracellular calcium levels.

In remarkable contrast, particulate Env delivery resulted in an increased calcium signal compared to soluble Env and lowered the required concentration for induction of the calcium signal. These findings are consistent with several studies that reported enhanced activation of B cells by multivalent antigens [45]. Several underlying mechanisms by which B cell activation is triggered are discussed in the literature [46]. A widely accepted model is the BCR clustering model involving actin remodeling and caveolin-dependent organization, which results in the formation of BCR clusters [47,48]. Accordingly, we observed clustered or punctuated distribution of BCRs upon activation with Env-particle conjugates, while activation with soluble Env showed very little of that punctuated pattern. Similar observations were made by Qin et al., who observed a punctuated distribution of BCRs upon activation with multivalent antigens, while monovalent antigens exhibited a more even distribution [49].

Surprisingly, both particle sizes showed comparable B cell activation, despite having major differences in their physicochemical characteristics like particle size, curvature, charge, ligand flexibility, and ligand density–all of which have been shown to affect multivalent interactions [36]. Obviously, the greatest difference is the size of the particles which corresponds to a higher curvature for the smaller SiNP_100_ particles. Hence, size can control the degree of multivalent interactions with BCRs [50]. Following binding, clathrin-coated pits may be formed, which could potentially favor endocytosis of particles below 200 nm because they would fit better in these pits [51,52]. Additionally, Env mobility may differ between particle sizes and affect binding. While Env was coupled to the 100 nm SiNPs via a short sulfo-SMCC linker, a linker with an ethylene glycol spacer was used for the SiNP_200_ particles to allow for higher flexibility. This in turn may increase the likelihood of receptor binding [53]. Together these differences may impact BCR engagement and activation. However, confocal images did not unveil any difference between activation caused by the two particle sizes.

Finally, we immunized mice to analyze the immunogenicity of our SiNP_100_-conjugates in vivo. We decided to use the traditional single-dose prime–boost method and the subcutaneous route as this has been shown to be particularly beneficial for nanoparticulate immunogens [54]. We chose MPLA as an adjuvant because it is a non-toxic version of lipopolysaccharide (LPS) that retains the immune-stimulatory properties of LPS as a potent TLR4-agonist but exhibits low toxicity [55,56]. Furthermore, MPLA has been shown to induce a favorable type 1 helper T cell (T_h_1) biased immune response in the context of HIV-1 VLPs and has been successfully used with stabilized Env trimers on the surface of liposomes [25,57]. In agreement with the findings of others, we found a higher and earlier immune response for the nanoparticle-presented Env [10,20,45,58]. The SiNP-coupled Env induced a serum reactivity comparable to the adjuvanted soluble protein at a lower dose and when no adjuvant was used. Thus, SiNPs provide a multiple dose sparing effect as they (i) reduce the amount of antigen, (ii) reduce the number of immunizations needed for a strong immune reaction and (iii) reduce the amount of adjuvant necessary for a strong immune reaction. An interesting aspect of mesoporous SiNPs is their intrinsic ability to act as an adjuvant [59,60]. Bulk SiNPs as used in our study may have a similar effect because mice that were immunized with Env coupled to SiNP_100_ without MPLA showed similar serum titers as mice that received soluble Env plus MPLA. Although not significant, our data on the avidity of the induced antibodies suggests earlier B cell maturation. Further investigation is warranted into which epitopes the elicited antibodies target and how strong and broad their neutralization capacity is.

## 5. Conclusions

In this study, we addressed critical design parameters required for efficient nanoparticulate delivery of Env-based immunogens. We covalently conjugated Env trimers in a specifically-oriented and stable manner to the surface of two differently sized silica nanoparticles with improved ligand density and attachment stability. Both particle formulations were superior to soluble Env in terms of cellular uptake by BMDCs and activation of B cells. We used the 100 nm SiNPs for investigation in a mouse immunization study due to their favorable colloidal stability and their potential of accumulating within B cell rich regions in the lymph node. In this in vivo study, we showed an early, strong, and sustained immune reaction to the SiNP_100_-Env particles, which was superior to the soluble, adjuvanted protein even at a lower dose.

In upcoming studies, we aim to further characterize the immune response to SiNP-coupled immunogens in terms of lymph node trafficking, GC formation, neutralization capacity of the induced response, mapping of the predominant epitopes as well as the quality and quantity of the induced T cell reaction. It will also be of interest, whether our SiNP-particle formulation can help to expand and guide low affinity germline precursors of bnAbs in vitro and in vivo in suitable animal models [61].

## Figures and Tables

**Table 1:** Overview of the physicochemical properties of particles before and after Env conjugation.

Particle Preparation	Hydrodynamic Diameter/nm	Polydispersity Index	Zeta Potential/mV
SiNP_200_	244 ± 2	0.085 ± 0.067	−6.6 ± 1.5
SiNP_200_-Env	371 ± 9	0.228 ± 0.096	−6.3 ± 5.7
SiNP_100_	102 ± 1	0.021 ± 0.014	−39.2 ± 2.1
SiNP_100_-Env	131 ± 2	0.045 ± 0.019	−9.6 ± 2.9

## Supplementary Materials

The following are available online at https://www.mdpi.com/article/10.3390/vaccines9060642/s1, Figure S1: SDS PAGE for all analyzed variants, Figure S2: Analysis of the influence of TCEP treatment to trimer integrity by Native PAGE. Figure S3: Nano DSF analysis. Figure S4: Optimization of reaction conditions for the attachment of Env to 200 nm SiNPs. Figure S5: Optimization of reaction conditions for the attachment of Env to 100 nm SiNPs. Figure S6: Nanoparticle associated Env uptake by BMDCs. Figure S7: Representative cytometry plots of the CD11c and CD86 phenotype of BMDC following activation. Figure S8: Percentage of CD11c and CD80, CD86 and MHCII positive cells. Figure S9: Comparison between Area under curve (AUC) and Effective Concentration 50 (EC50) values. Figure S10: Specificity of the coupling via the N-terminal tag. Figure S11: SiNP100-Env showed colloidal stability over a period of 12 weeks at 4°C. Figure S12: Release of Env adsorbed to SiNP200 particles. Table S1: Exemplary expression yields as purified by Ni-affinity chromatography from 293Expi expression supernatants.
