# Supplementary material for: Augmenting the Immune Response against a Stabilized HIV-1 Clade C Envelope Trimer by Silica Nanoparticle Delivery
Source: Vaccines (Basel). 2021 Jun 11;9(6):642. doi: 10.3390/vaccines9060642 (PMC8230641; doi:10.3390/vaccines9060642)
Supplement: Supplementary file 1 [file vaccines-09-00642-s001.zip › vaccines-1225503-supplementary.pdf]

Supplementary figures and tables

Augmenting the immune response against a stabilized HIV-1 Clade C envelope trimer by silica nanoparticle delivery

David Peterhoff[1]*, Stefanie Thalhauser[2], Jan M. Sobczak[3,4], Mona O. Mohsen[3,4], Christoph Voigt[1], Nicole Seifert[1], Patrick Neckermann[1], Alexandra Hauser[1], Song Ding[5], Quentin Sattentau[6], Martin F. Bachmann[3,4,7], Miriam Breunig[2] and Ralf Wagner[1,8]*

1 Institute of Medical Microbiology and Hygiene, University of Regensburg, 93053 Regensburg, Germany; david.peterhoff@ur.de (D.P.), ralf.wagner@ur.de (R.W.), christoph.voigt@stud.uni-regensburg.de (C.V.), nicole.seifert@uni-hohenheim.de (N.S.), patrick.neckermann@klinik.uni-regensburg.de (P.N.), alexandra.hauser@klinik.uni-regensburg.de (A.H.)

2 Institute of Pharmaceutical Technology, University of Regensburg, 93053 Regensburg, Germany; stefanie.thalhauser@gmail.com (S.T.), miriam.breunig@chemie.uni-regensburg.de (M.Br.)

3 Department for BioMedical Research, University of Bern, 3010 Bern, Switzerland; jan.sobczak@dbmr.unibe.ch (J.S.), mona.mohsen@dbmr.unibe.ch (M.M.), mar-tin.bachmann@dbmr.unibe.ch (M.Ba.)

4 Department of Immunology RI, University Hospital Bern, 3010 Bern, Switzerland

5 EuroVacc Foundation, 1002 Lausanne, Switzerland; song.ding@eurovacc.org (S.D.)

6 Sir William Dunn School of Pathology, University of Oxford, Oxford, United Kingdom; quentin.sattentau@path.ox.ac.uk (Q.S.)

7 Jenner Institute, Nuffield Department of Medicine, University of Oxford, Oxford, United Kingdom

8 Institute of Clinical Microbiology and Hygiene, University Hospital Regensburg, Regensburg, Germany; ralf.wagner@ur.de (R.W.)

* Correspondence: david.peterhoff@ur.de and ralf.wagner@ur.de; Tel.: +49 941 94416459 (D.P.), +49 941 9446452 (R.W.)

Table S1: Exemplary expression yields as purified by Ni-affinity chromatography from 293Expi expression supernatants

Variant	Expression yield per liter cell culture / mg
BG505-SOSIP	3.12
BG505-NFL	3.76
BG505-NtCC	4.19
ConC-SOSIP	18.30
ConC-NFL	24.41
ConC-NtCC	15.22

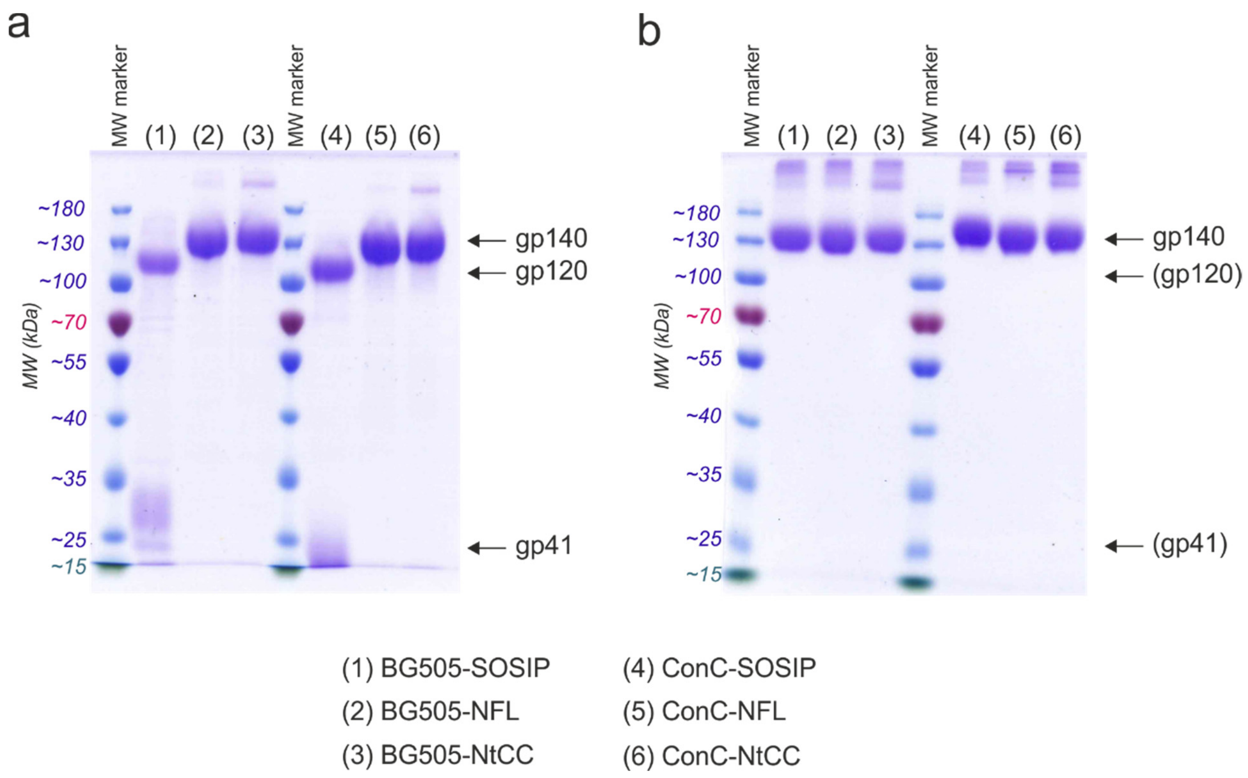

Fig. S1: SDS PAGE for all analyzed variants. 10% acrylamide gels were used. (**a**) reducing conditions; (**b**) non-reducing conditions. MW marker indicates sizes of protein bands. 5 µg protein was loaded per lane.

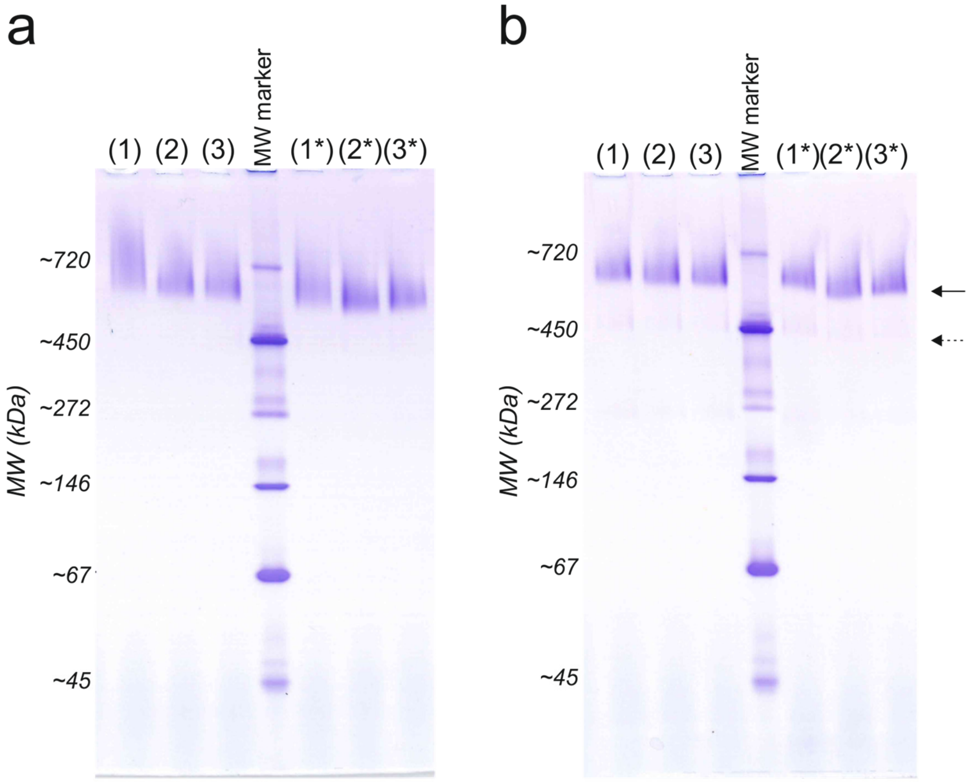

(1) BG505-SOSIP
(2) BG505-NFL
(3) BG505-NtCC

(4) ConC-SOSIP
(5) ConC-NFL
(6) ConC-NtCC

* TCEP treated

Fig. S2: Analysis of the influence of TCEP treatment to trimer integrity by Native PAGE. To mimic the procedure during coupling of the protein to SiNPs, Env was treated with 1 mM TCEP for 1 h at ambient temperature, subsequently diluted to 0.5 mM TCEP and further incubated overnight at 4°C. 2.5 µg of protein was loaded on each lane of the 4–16% acrylamide native PAGE. ConC variants (**a**) and BG505 variants (**b**) were analyzed. Solid line arrow indicates trimer bands; dashed line arrow indicates dimer bands; MW marker indicates size of protein bands; asterisks mark TCEP treated samples.

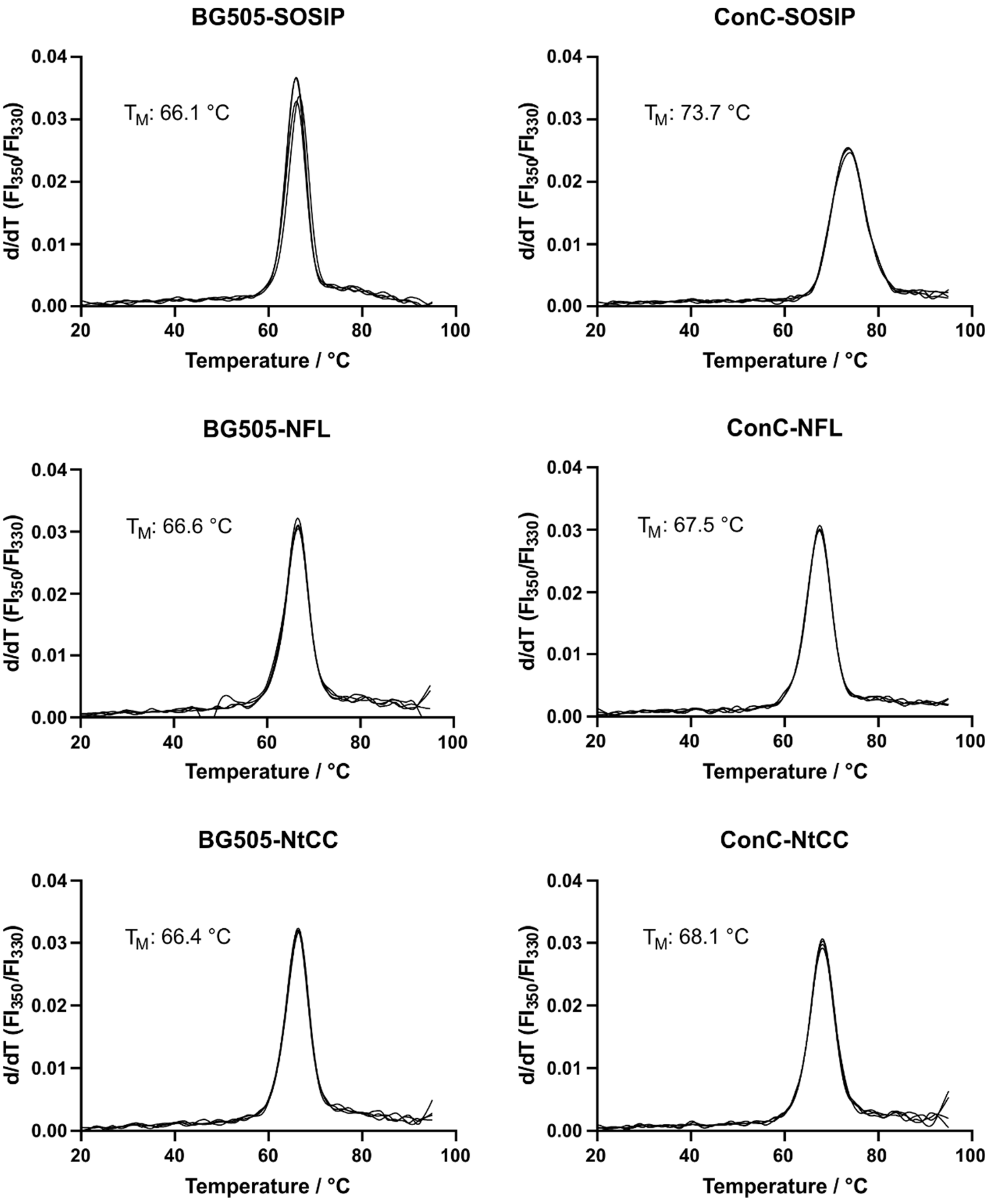

Fig. S3: Nano DSF analysis. 75 ng/mL protein in PBS was used for all measurements. Results from three replicate runs are shown. FI, Fluorescence intensity at 330 nm and 350 nm; TM, mean melting temperature from the three replicates.

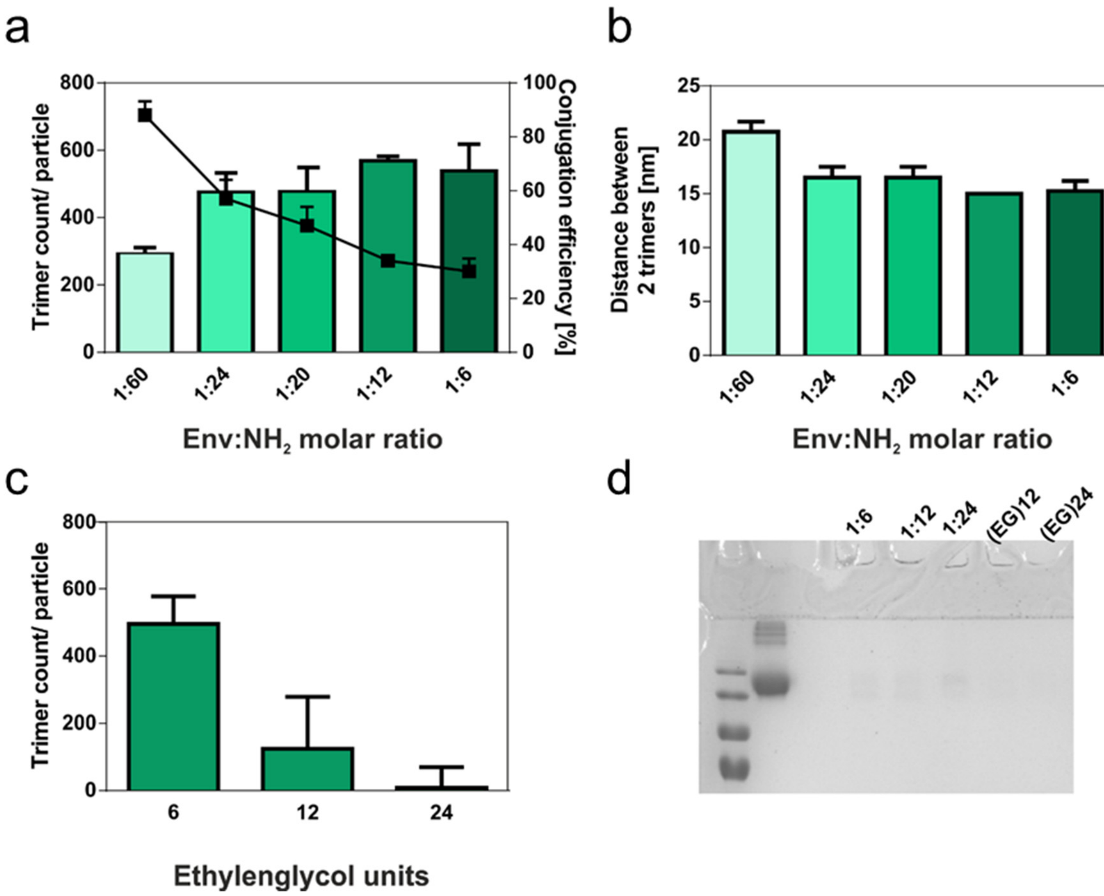

Fig. S4: Optimization of reaction conditions for the attachment of Env to 200 nm SiNPs. The number of trimers per particle (**a**) and distances (**b**) increased with increasing Env to NH_2 molar ratio corresponding to an increasing amount of Env added to the reaction, while conjugation efficiency decreased using SM$(EG)_6$ as linker. At molar ratios of 1:12 a sufficient Env loading of about 600 trimers per particle with acceptable conjugation efficiency of about 40% was achieved and was used for cell experiments. Applying a prolonged linker with a larger ethyylenglycol linker did result in lower Env attachment (**c**). Representative SDS PAGE of selected formulations. Lane 1: ladder, Lane 2: soluble Env, Lane 3: SiNPs, Lane 4-6: Env attached to SiNPs via SM$(EG)_6$ linker at different ratios. Lane 7+8: Env attached to SiNPs via different linkers. Only soluble Env was detected in the gel after Coomassie staining (**d**). Results are presented as mean ± standard deviation (n=4 samples).

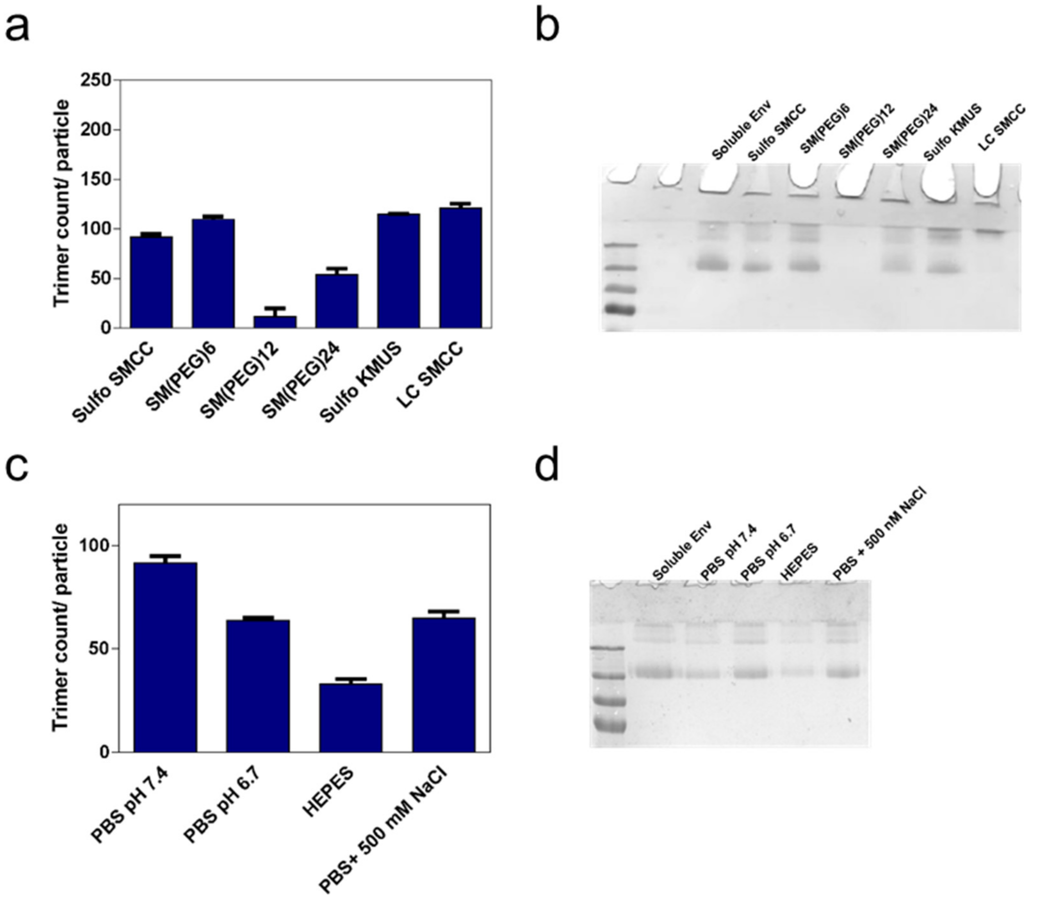

Fig. S5: Optimization of reaction conditions for the attachment of Env to 100 nm SiNPs. For optimization of conjugation to 100 nm BG505 NFL-NtCC variants were used at an Env to NH_2 molar ratio of 1:3 Trimers per particle (**a**) after conjugation using Sulfo SMCC, SM(PEG)$_6$, SM(PEG)$_{12}$, SM(PEG)$_{24}$, Sulfo KMUS, LC SMCC. (**b**) SDS PAGE of BG505 NtCC attached to 100 nm SiNPs. BG505 amount was adjusted to 2µg. Lane 1: ladder, lane 2: SiNPs, lane 3: BG505 NtCC, lane 4: SiNP-(SulfoSMCC)BG505-NtCC, lane 5: SiNP-(PEG$_6$)BG505-NtCC, lane 6: SiNP-(PEG$_{12}$)BG505-NtCC, lane 7: SiNP-(PEG$_{24}$)BG505-NtCC, lane 8: SiNP-(Sulfo KMUS)BG505-NtCC, lane 9: SiNP-(LC SMCC)BG505-NtCC. SDS PAGE indicated strong non-specific adsorption during conjugation reaction. Among the applied linkers sulfo SMCC showed the lowest non-specific adsorption and was thus used for the subsequent experiments. (**c**) To decrease non-specific adsorption maleimide to thiol reaction was conducted in different media. Highest loading was achieved with PBS 7.4 while HEPES resulted in a decreased loading. (**d**) SDS PAGE of Env attached to 100 nm particles in different media. Coomassie staining indicated that with PBS 7.4 non-specific adsorption was reduced to about 20%. Hence, for subsequent experiments sulfo SMCC linker was used and as coupling medium PBS with pH 7.4 was used. Results are presented as mean ± standard deviation (n=4 samples).

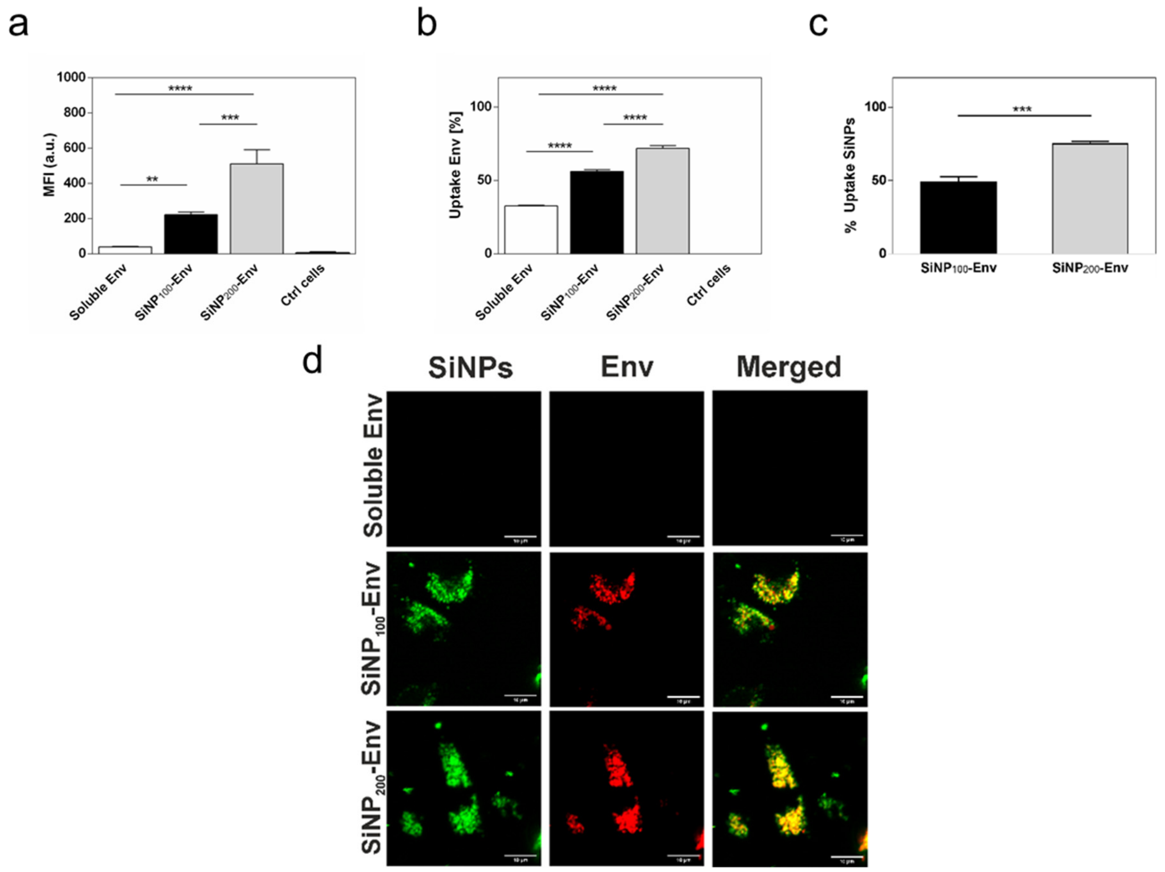

Fig. S6: Nanoparticle associated Env uptake by BMDCs. FACS analysis of the uptake of Env attached to SiNPs compared to the corresponding amount of soluble protein expressed as (**a**) mean fluorescence intensity (MFI) and (**b**) percentage of uptake. (**c**) Uptake of 100 nm SiNPs and 200 nm SiNPs by BMDCs. Results are presented as mean ± standard deviation (n=3 samples, levels of statistical significance are indicated as **p = 0.01, ***p = 0.001 and ****p = 0.0001). (**d**) CLSM analysis of the uptake of Env attached to SiNPs compared to soluble protein. Co-localization of SiNPs (green) and Env (red) was indicated by the yellow spots of the merged channels (scale bar=10 μm).

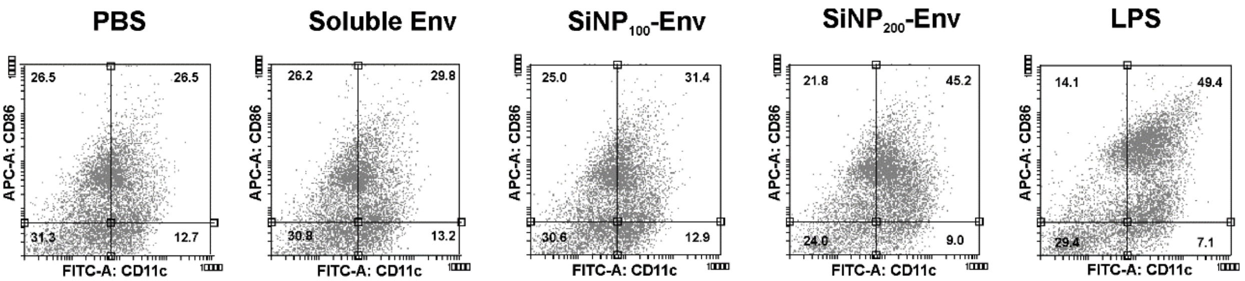

Fig. S7: Representative cytometry plots of the CD11c and CD86 phenotype of BMDC following activation.

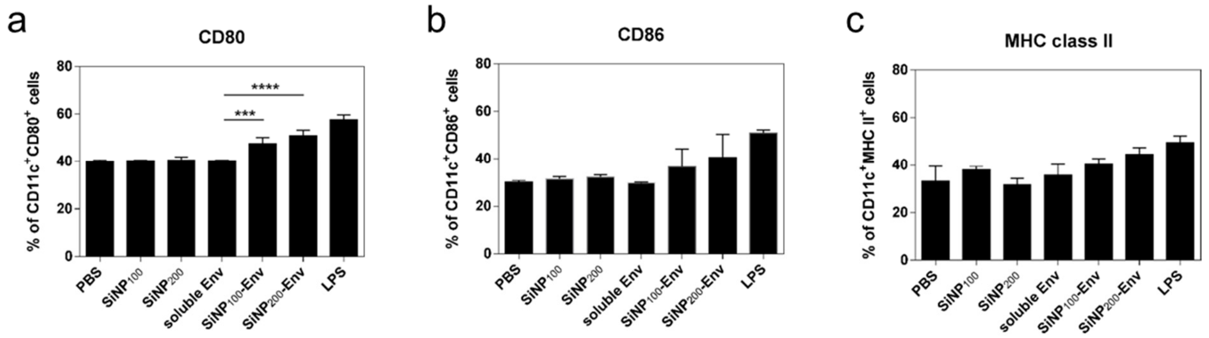

Fig. S8: Percentage of CD11c and CD80, CD86 and MHCII positive cells. (**a**)–(**c**) after stimulation of BMDCs for 16 hours. A slight increase of the amount of stimulated BMDCs was induced by a particulate Env presentation. Results are presented as mean ± standard deviation (n=3 samples, levels of statistical significance are indicated as *** p=0.001 and **** p=0.0001).

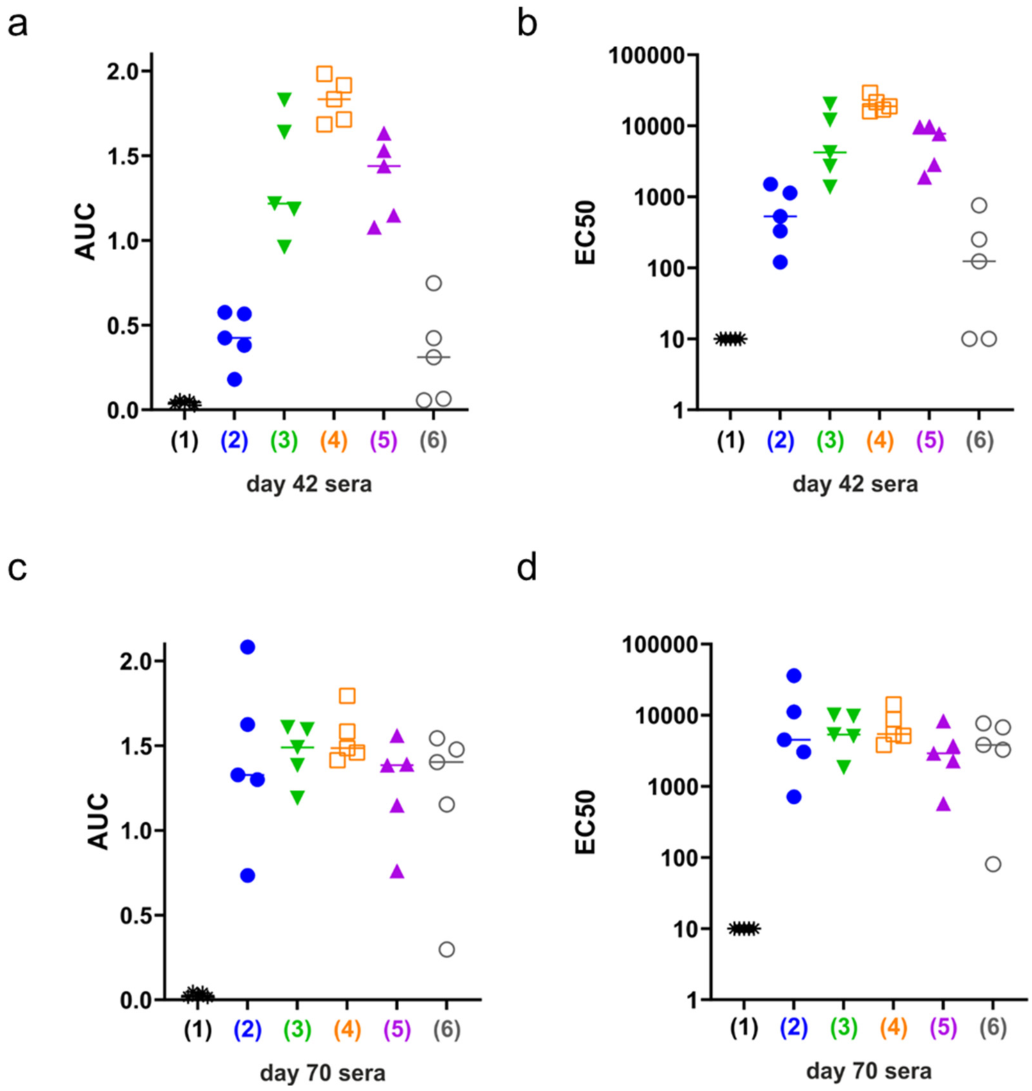

Fig. S9: Comparison between Area under curve (AUC) and Effective Concentration 50 (EC50) values. Results from the ELISA serum titration of each mouse, grouped per immunization group. (**a**)–(**b**) AUC and EC50 for bleed 3. (**c**)–(**d**) AUC and EC50 for bleed 4. Lines indicate the median for each group. Groups: (1), $SiNP_{100}$; (2), Env; (3), Env mixed with $SiNP_{100}$; (4), Env coupled to $SiNP_{100}$; (5), Env coupled to $SiNP_{100}$ at low dose; (6) Env coupled to $SiNP_{100}$ without adjuvant.

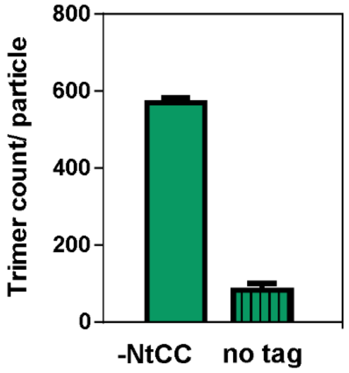

Fig. S10: Specificity of the coupling via the N-terminal tag. Env conjugation to $SiNP_{200}$ particles resulted in about 580 trimers per particle using Env with an N-terminal cysteine tag (NtCC), while Env without an N-terminal tag resulted only in 70 trimers per particle, indicating that the conjugation was formed between maleimides on the particle surface and thiols of the cysteine-tag. Results are presented as mean ± standard deviation (n=4 samples).

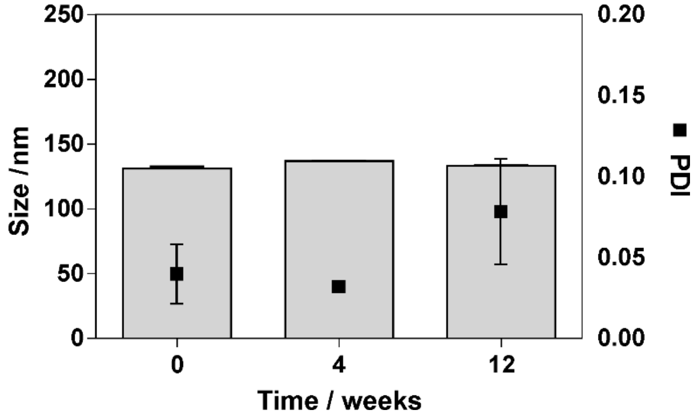

Fig. S11: $SiNP_{100}$-Env showed colloidal stability over a period of 12 weeks at 4°C. Results are presented as mean ± standard deviation (n=3 measurements).

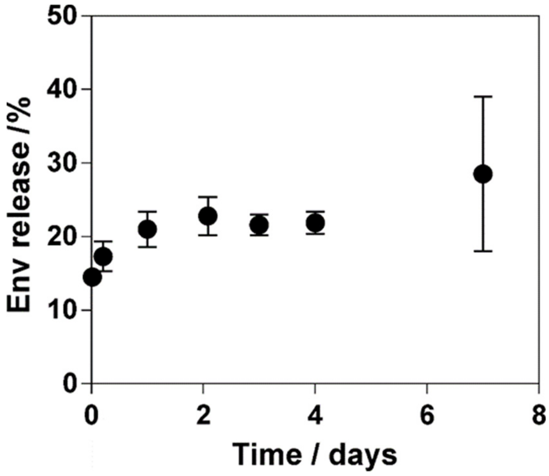

Fig. S12: Release of Env adsorbed to $SiNP_{200}$ particles. After an initial burst of about 15%, release slowed down reaching 30% after seven days in PBS at 37°C. Results are presented as mean ± standard deviation (n=3 samples).
